# Dombi power partitioned Heronian mean operators of *q*-rung orthopair fuzzy numbers for multiple attribute group decision making

**DOI:** 10.1371/journal.pone.0222007

**Published:** 2019-10-22

**Authors:** Yanru Zhong, Hong Gao, Xiuyan Guo, Yuchu Qin, Meifa Huang, Xiaonan Luo

**Affiliations:** 1 Guangxi Key Laboratory of Intelligent Processing of Computer Images and Graphics, Guilin University of Electronic Technology, Guilin, PR China; 2 School of Computing and Engineering, University of Huddersfield, Huddersfield, United Kingdom; 3 School of Mechanical and Electrical Engineering, Guilin University of Electronic Technology, Guilin, PR China; Shandong University of Science and Technology, CHINA

## Abstract

In this paper, a set of Dombi power partitioned Heronian mean operators of *q*-rung orthopair fuzzy numbers (*q*ROFNs) are presented, and a multiple attribute group decision making (MAGDM) method based on these operators is proposed. First, the operational rules of *q*ROFNs based on the Dombi t-conorm and t-norm are introduced. A *q*-rung orthopair fuzzy Dombi partitioned Heronian mean (*q*ROFDPHM) operator and its weighted form are then established in accordance with these rules. To reduce the negative effect of unreasonable attribute values on the aggregation results of these operators, a *q*-rung orthopair fuzzy Dombi power partitioned Heronian mean operator and its weighted form are constructed by combining *q*ROFDPHM operator with the power average operator. A method to solve MAGDM problems based on *q*ROFNs and the constructed operators is designed. Finally, a practical example is described, and experiments and comparisons are performed to demonstrate the feasibility and effectiveness of the proposed method. The demonstration results show that the method is feasible, effective, and flexible; has satisfying expressiveness; and can consider all the interrelationships among different attributes and reduce the negative influence of biased attribute values.

## 1. Introduction

Multi-attribute group decision making (MAGDM) is a process of choosing the best alternative in complex scenarios by using a group of decision makers to evaluate the values of multiple attributes of all alternatives synthetically. In this process, the primary task is to accurately express the attribute values, and fuzzy sets are regarded as effective tools for such expression. To date, over twenty different types of fuzzy sets have been presented within academia [1]. Among them, Zadeh’s fuzzy set (FS) [2] is a well-known type of fuzzy set that uses degree of membership to quantify degree of satisfaction. However, this fuzzy set cannot express nonmembership and hesitancy degree. Atanassov [3] proposed the intuitionistic FS (IFS) to overcome this shortcoming by adding a nonmembership degree; thus, the hesitancy function can be expressed as one minus the sum of the membership and nonmembership degrees. Because IFSs can describe more complex fuzzy information than FSs, many research topics regarding them have been presented, such as the operational rules of intuitionistic fuzzy numbers (IFNs) [4], aggregation operators of IFNs [5], intuitionistic fuzzy preference relations [6], rules of intuitionistic fuzzy calculus [7], and MAGDM methods based on IFSs [8]. Although IFSs have shown great potential in MAGDM, their application range is limited by their ability to express fuzzy information, i.e., the sum of membership and nonmembership degrees should be within the range of 0 to 1. To address this issue, Yager [9] proposed the theory of Pythagorean fuzzy sets (PFSs), in which the condition is extended to the sum of the squares of the membership and nonmembership degrees falling within the range of 0 to 1. Because they have greater expressiveness than IFSs, PFSs have also received much attention from researchers. For example, Yager and Abbasov [10] investigated the relationships among Pythagorean fuzzy numbers (PFNs); Peng and Yang [11] proposed division and subtraction operations on PFSs; Dick et al. [12] developed interpretations of complex-valued Pythagorean membership grades; Liang et al. [13] proposed a new model of three-way decisions based on PFSs.

Recently, to further improve the expressiveness of PFS, Yager [14] presented the concept of the generalized orthopair fuzzy set, i.e., the *q*-rung orthopair fuzzy set (*q*ROFS), in which the membership and nonmembership degrees satisfy the condition that the sum of their *q*-th powers lies within the range of 0 to 1. Obviously, IFSs and PFSs are special cases of *q*ROFSs with *q* = 1 and *q* = 2. This feature makes the expressiveness of *q*ROFSs more powerful than that of IFSs and PFSs by assigning an appropriate value to *q*. For example, suppose that a decision maker is influenced by personal wishes or the surrounding environment and assigns special attribute values to product quality. The attribute values have a membership degree of 0.8 and a nonmembership degree of 0.8, i.e., (0.8,0.8). Obviously, neither an IFS nor a PFS can be applied in this case because 0.8+0.8>1 and 0.8^2^+0.8^2^>1. However, the attribute values can be expressed using a *q*ROFS by increasing the value of the parameter *q* (*q*≥4). It is worth noting that as the parameter *q* increases, the space of acceptable orthopairs increases, and more orthopairs will satisfy the bounding constraint. Therefore, *q*ROFSs are more flexible and more suitable for describing fuzzy information by dynamically adjusting the value of the parameter *q*. The subject of *q*ROFSs has received extensive attention in recent years. Various research topics regarding *q*ROFSs are gaining importance within academia, such as the score function of *q*ROFNs [15, 16], distance measures of *q*ROFNs [16, 17], correlation and correlation coefficients of *q*ROFSs [18], and extensions of *q*ROFSs [19].

To solve MAGDM problems, there are generally two groups of methods: conventional methods, such as TOPSIS, VIKOR, and ELECTRE, and methods based on aggregation operators. Aggregation operators can solve MAGDM problems more effectively than traditional approaches because they can provide comprehensive values and then give the ranking results, while conventional methods can only generate rankings. Aggregation operators are usually considered in terms of operational rules and functions: (1) For operational rules, note that some aggregation operators are special cases of members in the t-norm (TN) and t-conorm (TC) families, and the Archimedean t-norm and t-conorm are the generalization of many TNs and TCs. To date, many operators and operational rules of *q*ROFSs correspond to specific types of TNs and TCs and their operational rules, such as the Archimedean Bonferroni mean operator [20], the Archimedean Muirhead mean operator [21] the Hamacher operational rules [22], and the Frank operational rules [23]. (2) Yager [24] proposed the power average (PA) operator, which is a new tool to aggregate input arguments by considering the relationships among the attribute values. It allows attribute values to support and reinforce each other and thus can reduce the negative influence of unreasonable arguments on the aggregation result. To consider the relationships among the aggregated arguments, more than twenty different aggregation operators of *q*ROFSs have been studied, such as weighted averaging (WA) and weighted geometric (WG) operators [25], Bonferroni mean (BM) and geometric Bonferroni mean (GBM) operators [26], power BM operators [27], partitioned BM and partitioned GBM operators [28],extended BM operators [29], Maclaurin symmetric mean (MSM) and geometric Maclaurin symmetric mean operators [30], partitioned MSM and power partitioned MSM operators [31], power MSM operators [32], Hamy mean operators [33], Muirhead mean (MM) and geometric MM operators [34], power MM operators [35], weighted point operators [36], Heronian mean (HM) operators [37,38], geometric HM operators [37], and partitioned HM (PHM) operators [38]. In the existing literature, Yu et al. [39] explained the advantages of HM operators over BM operators in detail. Although these two aggregation operators can consider the interrelationships among the aggregated parameters, they can only address decision-making problems in which interrelationships occur only among attributes in the same partition, not among attributes in different partitions. Liu et al. [38] proposed partitioned HM operators based on qROFSs, and Liu et al. [40] proposed partitioned HM operators based on linguistic intuitionistic fuzzy sets to overcome this shortcoming by dividing the attribute values into several different sorts, such that multiple attributes in different classes are unrelated.

The recently proposed Dombi t-conorm and Dombi t-norm (DTT) [41], which are special types of the Archimedean t-norm and t-conorm (ATT), are powerful tools for information aggregation and have been applied to the aggregation of IFSs [42], hesitant fuzzy sets [43], and single-valued neutrosophic information [44]. However, they have not yet been applied to the aggregation of *q*ROFSs. It is interesting to extend the operational rules of *q*ROFNs based on the DTT. In addition, there is no aggregation operator that combines the PA operator and the partitioned HM operator to reflect the interrelationships among the input arguments and reduce the impact of some evaluation values provided by decision makers that are too high or too low due to lack of time and prior experience. It is also interesting to extend the PA operator and the partitioned HM operator to *q*ROFNs based on the DTT. Motivated by these considerations, a *q*-rung orthopair fuzzy Dombi power partitioned HM operator and its weighted form are presented in this paper, and a MAGDM method based on them is proposed.

The remainder of the paper is organized as follows. Section 2 briefly recalls some basic concepts of *q*-rung orthopair fuzzy sets, the DTT, the PA operator, the PHM operator and the operational rules of *q*ROFNs based on the DTT. Section 3 presents a set of operators for *q*ROFNs. Section 4 proposes a novel MAGDM method based on the presented operators. Section 5 provides a practical example, a set of experiments, qualitative comparisons, quantitative comparisons and further comparative analysis. The last section summarizes the paper.

## Preliminaries

### 2.1 *q*ROFSs

**Definition 1** [14]. A *q*ROFS *Q* in a finite universe of discourse X is:

$$Q=\left\{\langle x,\mu_{Q}(x),v_{Q}(x)\rangle |x\in X\right\}$$ (1)

where *μ*_*Q*_: X → [0, 1] denotes the degree of membership of the element *x* ∈ X to the set *Q* and *ν*_*Q*_: X → [0, 1] denotes the degree of nonmembership of the element *x* ∈ X to the set *Q*, with the condition that 0 ≤ (*μ*_*Q*_(*x*)^*q*^ + *ν*_*Q*_(*x*)^*q*^) ≤ 1 (*q* = 1, 2, 3, …). The degree of hesitancy (indeterminacy) of the element *x*∈ X to the set *Q* is:

πQ(x)=(1−(μQ(x))q−(vQ(x))q)1q (2)

For convenience, a pair (*μ*_*Q*_(*x*), *ν*_*Q*_(*x*)) is called a *q*ROFN [14] and denoted Θ = (*μ*, *v*). To compare two *q*ROFNs, their scores and accuracies must be calculated. The following is the definitions of the score of a *q*ROFN and the accuracy of a *q*ROFN.

**Definition 2** [14]. Let Θ = (*μ*, *v*) be a *q*ROFN. Then, the score of Θ is:

$$S(\Theta )=\mu^{q}-v^{q}$$ (3)

where -1 ≤ *S*(Θ) ≤1.

**Definition 3** [14]. Let Θ = (*μ*, *v*) be a *q*ROFN. Then, the accuracy of Θ is:

$$A(\Theta )=\mu^{q}+v^{q}$$ (4)

where 0 ≤ A(Θ) ≤1.

A method for comparing *q*ROFNs based on *S*(Θ) and A(Θ)) is presented in [14]. The following is the definition of the method.

**Definition 4** [14]. Let Θ_1_ = (*μ*_1_, *v*_1_) and Θ_2_ = (*μ*_2_, *v*_2_) be two arbitrary *q*ROFNs; let *S*(Θ_1_) and *S*(Θ_2_) be the scores of Θ_1_ and Θ_2_, respectively; and let A(Θ_1_) and A(Θ_2_) be the accuracies of Θ_1_ and Θ_2_, respectively. Then,

1. If *S*(Θ_1_)> *S*(Θ_2_), then Θ_1_ > Θ_2_;
2. If *S*(Θ_1_) = *S*(Θ_2_), then
(1) If A(Θ_1_) > A(Θ_2_), then Θ_1_ > Θ_2_;
(2) If A(Θ_1_) = A(Θ_2_), then Θ_1_ = Θ_2_.

**Definition 5** [17]. Let Θ_1_ = (*μ*_1_, *v*_1_) and Θ_2_ = (*μ*_2_, *v*_2_) be two arbitrary *q*ROFNs; then, the Minkowski-type distance between Θ_1_ and Θ_2_ is given by:

$$d(\Theta_{1},\Theta_{2})={\left(\frac{1}{2}{\left|\mu_{1}-\mu_{2}\right|}^{p}+\frac{1}{2}{\left|v_{1}-v_{2}\right|}^{p}\right)}^{1/p}(p>1)$$ (5)

### 2.2 Dombi t-norm and conorm

In the following, a new operational rule of *q*ROFNs is introduced based on the DTT [41] to generate a t-norm (TN) and t-conorm (TC):

$$D(x,y)=\phi^{-1}(\phi (x)+\phi (y))$$ (6)

(1) If *f* (*x*) is a monotonically increasing function such that:
  $$f(x):(0,1]\to R^{+};\;f^{‐1}(x):R^{+}\to (0,1];\;\lim_{x\to \infty }f{\left(x\right)}^{-1}=0;\;f^{-1}(0)=1,$$
  then the TN *T* can be defined as *T* (*x*, *y) = f* ^-1^ (*f* (*x*) *+ f* (*y*)).
(2) If *g* (*x*) is a monotonically decreasing function such that:
  $$g(x):(0,1]\to R^{+};\;g^{‐1}(x):R^{+}\to (0,1];\;\lim_{x\to \infty }g{\left(x\right)}^{-1}=1;\;g^{-1}(0)=0,$$
  then the TC *S* can be defined as *S* (*x*, *y*) *= g*^-1^ (g(*x*) *+ g*(*y*)). According to [45], the relationship of *f*(*x*) and *g*(*x*) is *f* (*x*) *= g*(1-*x*).

**Definition 6** [41]. Let *λ* be a positive real number and *x*, *y*∈ [0, 1]. The DTT and their additive generators are defined as follows:

TD,λ(x,y)=f−1(f(x)+f(y))=(11+((1−xqxq)λ+(1−yqyq)λ)1λ)1q (7)

SD,λ(x,y)=g−1(g(x)+g(y))=(1−11+((xq1−xq)λ+(yq1−yq)λ)1λ)1q (8)

$$f(t)={\left(\frac{1-t^{q}}{t^{q}}\right)}^{\lambda },g(t)={\left(\frac{t^{q}}{1-t^{q}}\right)}^{\lambda }$$ (9)

Then, the following can be obtained:

f‐1(t)=(11+t1λ)1q,g−1(t)=(t1λ1+t1λ)1q (10)

### 2.3 Operational rules of *q*ROFNs based on the DTT

On the basis of the DTT, a set of operational rules of *q*ROFNs can be established as follows:

**Definition 7**. Let Θ = (*μ*, *v*), Θ_1_ = (*μ*_1_, *v*_1_) and Θ_2_ = (*μ*_2_, *v*_2_) be three arbitrary *q*ROFNs, and let *δ* and *τ* be two arbitrary positive real numbers. Then, the sum, product, multiplication and power operations between *q*ROFNs based on *T*_*D*, *λ*_ (*x*, *y) = f* ^-1^ (*f* (*x*) *+ f* (*y*)) and *S*_*D*, *λ*_ (*x*, *y*) *= g*^-1^ (g(*x*) *+ g*(*y*)) can be defined as follows, respectively:

Θ1⊕Θ2=(g−1(g(μ1)+g(μ2),f−1(f(v1)+f(v2))=((1−11+((μ1q1−μ1q)λ+(μ2q1−μ2q)λ)1λ)1q,(11+((1−v1qv1q)λ+(1−v2qv2q)λ)1λ)1q) (11)

Θ1⊗Θ2=(f−1(f(μ1)+f(μ2)),g−1(g(v1)+g(v2)))=((11+((1−μ1qμ1q)λ+(1−μ2qμ2q)λ)1λ)1q,(1−11+((v1q1−v1q)λ+(v2q1−v2q)λ)1λ)1q) (12)

δΘ=(g−1(δg(μ)),f−1(δf(v)))=((1−11+(δ(μq1−μq)λ)1λ)1q,(11+(δ(1−vqvq)λ)1λ)1q) (13)

Θτ=(f−1(τf(μ)),g−1(τg(v)))=((11+(τ(1−μqμq)λ)1λ)1q,(1−11+(τ(vq1−vq)λ)1λ)1q) (14)

By using Eqs (11)–(14), it is easy to obtain the following rules:

$$\Theta_{1}⊕\Theta_{2}=\Theta_{2}⊕\Theta_{1}$$ (15)

$$\Theta_{1}⊗\Theta_{2}=\Theta_{2}⊗\Theta_{1}$$ (16)

$$\delta \left(\Theta_{1}⊕\Theta_{2}\right)=\delta \Theta_{1}⊕\delta \Theta_{2}$$ (17)

δΘ⊕τΘ=(δ+τ)Θ (18)

$$\Theta_{{}_{1}}^{\delta }⊗\Theta_{{}_{2}}^{\delta }={\left(\Theta_{1}⊗\Theta_{2}\right)}^{\delta }$$ (19)

$$\Theta^{\delta }⊗\Theta^{\tau }=\Theta^{\tau +\delta }$$ (20)

For the proofs of Eqs (15)–(20), please refer to Appendix A.

### 2.4 HM operator and PA operator

**Definition 8** [46]. Let *x*_*i*_ (*i* = 1, 2, …, *n*) be a series of crisp numbers. If

$$HM^{a,b}(x_{1},x_{2},\ldots ,x_{n})={\left(\frac{2}{n(n+1)}\sum_{i=1}^{n}\sum_{j=i}^{n}x_{i}^{a}x_{j}^{b}\right)}^{\frac{1}{a+b}}$$ (21)

where *a*, *b* ≥ 0, then *HM*^*a*,*b*^ is called the HM operator.

**Definition 9** [46]. Let X_*hi*_ (*i* = 1, 2, …, *n*) be a collection of arguments that is partitioned into *d* distinct sorts *P*_*1*_, *P*_*2*_, *…*,*P*_*d*_, where *P*_*h*_ = {X_*h1*_, X_*h2*_,…, X_*h|Ph|*_} (*h* = 1,2,…,*d*) and $\sum_{h=1}^{d}\left|P_{h}\right|=mand\left|P_{h}\right|$ denotes the cardinality of *P*_*h*_. For any *a*, *b* ≥ *0*, the aggregation function

$$PHM^{a,b}(\chi_{1},\chi_{2},\ldots ,\chi_{n})=\frac{1}{d}\left(\sum_{h=1}^{d}{\left(\frac{2}{\left|P_{h}\right|(\left|P_{h}\right|+1)}\sum_{i=1}^{\left|P_{h}\right|}\sum_{j=i}^{\left|P_{h}\right|}\chi_{hi}^{a}\chi_{hj}^{b}\right)}^{\frac{1}{a+b}}\right)$$ (22)

is called the PHM operator.

**Definition 10** [24]. Let *a*_*i*_ (*i* = 1, 2, …, *n*) be a collection of nonnegative real numbers. Then

$$PA(a_{1},a_{2},\ldots ,a_{n})=\frac{\sum_{i=1}^{n}\left(\left(1+T(a_{i})\right)a_{i}\right)}{\sum_{i=1}^{n}\left(1+T(a_{i})\right)}$$ (23)

is called the power average (PA) operator, where

$$T(a_{i})=\sum_{\begin{array}{l} j=1\\ j\neq i \end{array}}^{n}Sup(a_{i},a_{j})$$ (24)

and *Sup* (*a*, *b*) denotes the support for *a* from *b*, which satisfies the following three conditions:

(1) *Sup*(*a*,*b*) = [0,1];
(2) *Sup*(*a*,*b*) = *Sup*(*b*,*a*);
(3) *Sup*(*a*,*b*)≥*Sup*(*x*,*y*), *if* |*a*−*b*|≤|*x*−*y*|

To simplify Eq (23), let *V*_*i*_ = 1 + *T*(*a*_*i*_) and $w_{i}=\frac{V_{i}}{\sum_{i=1}^{n}V_{i}}$; then

$$PA(a_{1},a_{2},\ldots ,a_{n})=\sum_{i=1}^{n}\left(w_{i}a_{i}\right)$$ (25)

## 3. Dombi power partitioned Heronian mean operators of *q*ROFNs

### 3.1 *q*-Rung orthopair fuzzy Dombi partitioned Heronian mean operators

In this section, PHM is extended to the *q*-rung orthopair fuzzy environment, and a *q*-rung orthopair fuzzy Dombi partitioned Heronian mean (*q*ROFDPHM) operator and a *q*-rung orthopair fuzzy Dombi weighted partitioned Heronian mean (*q*ROFDWPHM) operator are presented. Their properties are explored.

**Definition 11**. Let {Θ_1_, Θ_2_, …, Θ_*n*_} (where Θ_*i*_ = (*μ*_*i*_, *v*_*i*_) (*i* = 1, 2, …, *n*) be a collection of *q*ROFNs (*q* = 1, 2, …) that is partitioned into *d* distinct sorts *P*_*1*_,*P*_*2*_,*…*,*P*_*d*_, where *P*_*h*_ = {Θ_*h1*_, Θ_*h2*_,…, Θ_*h|Ph|*_} (*h* = 1, 2, …, *d*) and |*P*_1_|+|*P*_2_|+…+|*P*_*d*_| = *n*. For any two real numbers *a* and *b* such that *a*, *b* ≥ *0* but *a* and *b* are not zero simultaneously, the *q*ROFDPHM operator is defined as:

$$qROFDPHM^{a,b}(\Theta_{1},\Theta_{2},\ldots ,\Theta_{n})=\frac{1}{d}{\left(\overset{d}{\underset{h=1}{⊕}}\left(\frac{2}{\left|P_{h}\right|(\left|P_{h}\right|+1)}\overset{\left|P_{h}\right|}{\underset{i=1}{⊕}}\overset{\left|P_{h}\right|}{\underset{j=i}{⊕}}\Theta_{hi}^{a}⊗\Theta_{hj}^{b}\right)\right)}^{\frac{1}{a+b}}$$ (26)

Based on Eqs (11)–(14) and (26), the following theorem is obtained:

**Theorem 1**. Let {Θ_1_, Θ_2_, …, Θ_*n*_} (where Θ_*i*_ = (*μ*_*i*_, *v*_*i*_) (*i* = 1, 2, …, *n*) be a collection of *q*ROFNs (*q* = 1, 2, …) that is partitioned into *d* distinct sorts *P*_*1*_,*P*_*2*_,*…*,*P*_*d*_, where *P*_*h*_ = {Θ_*h1*_, Θ_*h2*_,…, Θ_*h|Ph|*_} (*h* = 1, 2, …, *d*) and |*P*_1_|+|*P*_2_|+…+|*P*_*d*_| = *n*; let *a* and *b* be two real numbers such that *a*, *b* ≥ *0* but *a* and *b* are not zero simultaneously, and let *λ* be a positive real number. Then, the aggregated value produced by *q*ROFDHM is still a *q*ROFN, and

qROFDPHMa,b(Θ1,Θ2,…,Θn)=((1−(1/(1+(1d∑h=1d(2(a+b)|ph|(|ph|+1)∑i=1|Ph|∑j=i|Ph|1(af(uhi)+bf(uhj))))1λ)))1q,(1/(1+(1d∑h=1d(2(a+b)|ph|(|ph|+1)∑i=1|Ph|∑j=i|Ph|1(ag(vhi)+bg(vhj))))1λ))1q) (27)

where $f(\mu_{hi})={\left(\frac{1-\mu_{{}_{hi}}^{q}}{\mu_{{}_{hi}}^{q}}\right)}^{\lambda },f(\mu_{hj})={\left(\frac{1-\mu_{{}_{hj}}^{q}}{\mu_{{}_{hj}}^{q}}\right)}^{\lambda },g(v_{hi})={\left(\frac{v_{{}_{hi}}^{q}}{1-v_{{}_{hi}}^{q}}\right)}^{\lambda }\;\mathrm{and}\;g(v_{hj})={\left(\frac{v_{{}_{hj}}^{q}}{1-v_{{}_{hj}}^{q}}\right)}^{\lambda }$.

For the proof of Theorem 1, please refer to Appendix B.

**Theorem 2 (Idempotency)**. Let {Θ_1_, Θ_2_, …, Θ_*n*_} (where Θ_*i*_ = (*μ*_*i*_, *v*_*i*_) (*i* = 1, 2, …, *n*) be a collection of *q*ROFNs (*q* = 1, 2, …), and let *a* and *b* be two real numbers such that *a*, *b* ≥ *0* but *a* and *b* are not zero simultaneously. If Θ_*i*_ = Θ = (*μ*, *v*) for all *i* = 1, 2, …, *n*, then

$$qROFDPHM^{a,b}(\Theta_{1},\Theta_{2},\ldots ,\Theta_{m})=\Theta$$ (28)

**Proof**.

Let *qROFDPHM*^*a*,*b*^(Θ_1_,Θ_2_,…,Θ_*n*_) = (*μ*_*α*_,*v*_*α*_). It is shown that

$$qROFDPHM^{a,b}(\Theta_{1},\Theta_{2},\ldots ,\Theta_{n})=(\mu ,v).$$

Since Θ_*hi*_ = Θ = (*μ*,*v*) and Θ_*hj*_ = Θ = (*μ*,*v*), we have:

μα=(1−(1/(1+(1d∑h=1d(2(a+b)|ph|(|ph|+1)∑i=1|Ph|∑j=i|Ph|1(af(μhi)+bf(μhj))))1λ)))1q=(1−(1/(1+1d∑h=1d2(a+b)|ph|(|ph|+1)∑i=1|Ph|∑j=i|Ph|1(af(μ)+bf(μ))1λ)))1q=(1−(1/(1+(1d∑h=1d(2(a+b)|ph|(|ph|+1)∑i=1|Ph|∑j=i|Ph|1(a+b)f(μ))))1λ)))1q=(1−(1/(1+(1d∑h=1d(1f(μ)))1λ)))1q=(1−(1/(1+1(f(μ))1λ)))1q=(1−1+μq)1q=μ

where $f(\mu_{hi})={\left(\frac{1-\mu_{{}_{hi}}^{q}}{\mu_{{}_{hi}}^{q}}\right)}^{\lambda },f(\mu_{hj})={\left(\frac{1-\mu_{{}_{hj}}^{q}}{\mu_{{}_{hj}}^{q}}\right)}^{\lambda },g(v_{hi})={\left(\frac{v_{{}_{hi}}^{q}}{1-v_{{}_{hi}}^{q}}\right)}^{\lambda }\;\mathrm{and}\;g(v_{hj})={\left(\frac{v_{{}_{hj}}^{q}}{1-v_{{}_{hj}}^{q}}\right)}^{\lambda }.$

Similarly, it can also be shown that *v*_*α*_ = *v*. Thus

$$qROFDPHM^{a,b}(\Theta_{1},\Theta_{2},\ldots ,\Theta_{n})=(\mu_{\alpha },v_{\alpha })=(\mu ,v),$$

which completes the proof of Theorem 2.

**Theorem 3 (Monotonicity)**. Let {Θ_1_, Θ_2_, …, Θ_*n*_} (where Θ_*i*_ = (*μ*_*i*_, *v*_*i*_)) (*i* = 1, 2, …, *n*) and {Θ_1_’, Θ_2_’, …, Θ_*n*_*’*} (where Θ_*i*_*’* = (*μ*_*i*_*’*, *v*_*i*_*’*)) (*i* = 1, 2, …, *n*) be two collections of *q*ROFNs (*q* = 1, 2 …), and let *a* and *b* be two real numbers such that *a*, *b* ≥ *0* but *a* and *b* are not zero simultaneously. If *μ*_*i*_ ≤ *μ*_*i*_*’* and *v*_*i*_ ≤ *v*_*i*_*’* for all *i* = 1, 2, …, *n*, then

$$qROFDPHM^{a,b}(\Theta_{1},\Theta_{2},\ldots ,\Theta_{n})\leq qROFDPHM^{a,b}(\Theta_{1}^{\prime },\Theta_{2}^{\prime },\ldots ,\Theta_{n}^{\prime })$$ (29)

**Proof**.

Let

$$\begin{array}{l} qROFDPHM^{a,b}(\Theta_{1},\Theta_{2},\ldots ,\Theta_{n})=(\mu_{\alpha },v_{\alpha })\\ qROFDPHM^{a,b}(\Theta_{1}^{\prime },\Theta_{2}^{\prime },\ldots ,\Theta_{n}^{\prime })=(\mu^{\prime },v^{\prime }) \end{array}$$

Since $\mu_{hi}\leq \mu_{hi}^{\prime }$ and $\mu_{hj}\leq \mu_{hi}^{\prime }$, *f*(*t*),*f*^−1^(*t*) are monotonically decreasing, and *g*(*t*),*g*^−1^(*t*) are monotonically increasing, it follows that

$$\frac{1-\mu_{hi}^{q}}{\mu_{hi}^{q}}\geq \frac{1-\mu_{hi}^{\prime q}}{\mu_{hi}^{\prime q}},\frac{1-\mu_{hj}^{q}}{\mu_{hj}^{q}}\geq \frac{1-\mu_{hj}^{\prime q}}{\mu_{hj}^{\prime q}}$$

Therefore,

$$\left(af(u_{hi})+bf(u_{hj}))\geq (af(u_{hi}^{\prime })+bf(u_{hj}^{\prime })\right)$$

$$\left(\frac{1}{d}\sum_{h=1}^{d}\left(\frac{2(a+b)}{\left|p_{h}\right|(\left|p_{h}\right|+1)}\sum_{i=1}^{\left|P_{h}\right|}\sum_{j=i}^{\left|P_{h}\right|}\frac{1}{\left(af(u_{hi})+bf(u_{hj})\right)}\right)\right)\leq \left(\frac{1}{d}\sum_{h=1}^{d}\left(\frac{2(a+b)}{\left|p_{h}\right|(\left|p_{h}\right|+1)}\sum_{i=1}^{\left|P_{h}\right|}\sum_{j=i}^{\left|P_{h}\right|}\frac{1}{\left(af(u_{hi})+bf(u_{hj})\right)}\right)\right)$$

and

$$\left(1+\frac{1}{d}\sum_{h=1}^{d}\left(\frac{2(a+b)}{\left|p_{h}\right|(\left|p_{h}\right|+1)}\sum_{i=1}^{\left|P_{h}\right|}\sum_{j=i}^{\left|P_{h}\right|}\frac{1}{\left(af(u_{hi})+bf(u_{hj})\right)}\right)\right)\leq \left(1+\frac{1}{d}\sum_{h=1}^{d}\left(\frac{2(a+b)}{\left|p_{h}\right|(\left|p_{h}\right|+1)}\sum_{i=1}^{\left|P_{h}\right|}\sum_{j=i}^{\left|P_{h}\right|}\frac{1}{\left(af(u_{hi})+bf(u_{hj})\right)}\right)\right)$$

$$\left(1/\left(1+\frac{1}{d}\sum_{h=1}^{d}\left(\frac{2(a+b)}{\left|p_{h}\right|(\left|p_{h}\right|+1)}\sum_{i=1}^{\left|P_{h}\right|}\sum_{j=i}^{\left|P_{h}\right|}\frac{1}{\left(af(u_{hi})+bf(u_{hj})\right)}\right)\right)\right)\geq \left(1/\left(1+\frac{1}{d}\sum_{h=1}^{d}\left(\frac{2(a+b)}{\left|p_{h}\right|(\left|p_{h}\right|+1)}\sum_{i=1}^{\left|P_{h}\right|}\sum_{j=i}^{\left|P_{h}\right|}\frac{1}{\left(af(u_{hi})+bf(u_{hj})\right)}\right)\right)\right)$$

Then

μα=(1−(1/(1+1d∑h=1d(2(a+b)|ph|(|ph|+1)∑i=1|Ph|∑j=i|Ph|1(af(uhi)+bf(uhj))))))1q≤(1−(1/(1+1d∑h=1d(2(a+b)|ph|(|ph|+1)∑i=1|Ph|∑j=i|Ph|1(af(uhi)+bf(uhj))))))1q=μ

Thus *μ*_*α*_≤*μ*′. Similarly, it can be proved that *v*_*α*_≥*v*′.

Thus, $qROFDPHM^{a,b}(\Theta_{1},\Theta_{2},\ldots ,\Theta_{n})=(\mu_{\alpha },v_{\alpha })\leq qROFDPHM^{a,b}(\Theta_{1}^{\prime },\Theta_{2}^{\prime },\ldots ,\Theta_{n}^{\prime })=(\mu ,v)$, which completes the proof of Theorem 3.

**Theorem 4 (Boundedness)**. Let {Θ_1_, Θ_2_, …, Θ_*n*_} (where Θ_*i*_ = (*μ*_*i*_, *v*_*i*_)) (*i* = 1, 2,…, *n*) be a collection of *q*ROFNs (*q* = 1, 2, …), and let a and b be two real numbers such that *a*, *b* ≥ *0* but *a* and *b* are not zero simultaneously. If Θ_*S*_ = (max(*μ*_*i*_), min(*v*_*i*_)) and Θ_*I*_ = (min(*μ*_*i*_), max(*v*_*i*_)), then

$$\Theta_{I}\leq qROFDPHM^{a,b}(\Theta_{1},\Theta_{2},\ldots ,\Theta_{n})\leq \Theta_{S}$$ (30)

**Proof**.

From Theorem 2, we have:

$$qROFDPHM^{a,b}(\Theta_{I},\Theta_{I},\ldots ,\Theta_{I})=\Theta_{I},qROFDPHM^{a,b}(\Theta_{S},\Theta_{S},\ldots ,\Theta_{S})=\Theta_{S}.$$

From Theorem 3, we have:

$$qROFDPHM^{a,b}(\Theta_{I},\Theta_{I},\ldots ,\Theta_{I})\leq qROFDPHM^{a,b}(\Theta_{1},\Theta_{2},\ldots ,\Theta_{n})\leq qROFDPHM^{a,b}(\Theta_{S},\Theta_{S},\ldots ,\Theta_{S}).$$

Therefore, it follows that Θ_*I*_≤*qROFDPHM*^*a*,*b*^(Θ_1_,Θ_2_,…,Θ_*n*_)≤Θ_*s*_ which completes the proof of Theorem 4.

The following are some special cases of the proposed *q*ROFDPHM operator:

(1) Special cases with respect to parameters *a* and *b*.
  1) When *b*→0, Eq (27) reduces to
    qROFDPHMa,0(Θ1,Θ2,…,Θn)=((1−(1/(1+(1d∑h=1d(2|ph|(|ph|+1)∑i=1|Ph|(|Ph|−i+1)⋅1f(uhi)))1λ)))1q,(1/(1+(1d∑h=1d(2|ph|(|ph|+1)∑i=1|Ph|(|Ph|−i+1)⋅1g(vhi)))1λ))1q) (31)
    which is a *q*-rung orthopair fuzzy Dombi partitioned heavy averaging operator.
  2) When *b*→0 and all the *q*ROFNs are partitioned into one sort (*d* = 1), then Eq (27) reduces to
    qROFDPHMa,0(Θ1,Θ2,…,Θn)=((1−(1/(1+(2n(n+1)∑i=1n(n−i+1)⋅1f(ui))1λ)))1q,(1/(1+(2n(n+1)∑i=1n(n−i+1)⋅1g(vi))1λ))1q) (32)
    which is a *q*-rung orthopair fuzzy Dombi heavy averaging operator.
  3) When *b*→0 and all the *q*ROFNs are partitioned into n sorts (*d* = n), then Eq (27) reduces to
    qROFDPHMa,0(Θ1,Θ2,…,Θn)=((1−(1/(1+(1n∑h=1n1f(uh))1λ)))1q,(1/(1+(1n∑h=1n1g(vh))1λ))1q) (33)
    which is a *q*-rung orthopair fuzzy Dombi generalized averaging operator.
  4) When *a*→0, then Eq (27) reduces to
    qROFDPHM0,b(Θ1,Θ2,…,Θn)=((1−(1/(1+(1d∑h=1d(2|ph|(|ph|+1)∑i=1|Ph|∑j=i|Ph|1f(uhj)))1λ)))1q,(1/(1+(1d∑h=1d(2|ph|(|ph|+1)∑i=1|Ph|∑j=i|Ph|1g(vhj)))1λ))1q) (34)
    which is a *q*-rung orthopair fuzzy Dombi partitioned heavy averaging operator.
  5) When *a* = *b =* 1, then Eq (27) reduces to
    qROFDPHM1,1(Θ1,Θ2,…,Θn)=((1−(1/(1+(1d∑h=1d(4|ph|(|ph|+1)∑i=1|Ph|∑j=i|Ph|1f(uhi)+f(uhj)))1λ)))1q,(1/(1+(1d∑h=1d(4|ph|(|ph|+1)∑i=1|Ph|∑j=i|Ph|1g(vhi)+g(vhj)))1λ))1q) (35)
    which is a *q*-rung orthopair fuzzy partitioned line HM operator.
(2) Some special cases with respect to parameter *q*.
  1) When *q* = 1, the *q*ROFDPHM operator reduces to an intuitionistic fuzzy Dombi PHM (IFDPHM) operator:
    qROFDPHMa,b(Θ1,Θ2,…,Θn)=((1−(1/(1+(1d∑h=1d(2(a+b)|ph|(|ph|+1)∑i=1|Ph|∑j=i|Ph|1(af(uhi)+bf(uhj))))1λ))),(1/(1+(1d∑h=1d(2(a+b)|ph|(|ph|+1)∑i=1|Ph|∑j=i|Ph|1(ag(vhi)+bg(vhj))))1λ))) (36)
    where $f(\mu_{hi})={\left(\frac{1-\mu_{hi}}{\mu_{hi}}\right)}^{\lambda },f(\mu_{hj})={\left(\frac{1-\mu_{hj}}{\mu_{hj}}\right)}^{\lambda },g(v_{hi})={\left(\frac{v_{hi}}{1-v_{hi}}\right)}^{\lambda }\;\mathrm{and}\;g(v_{hj})={\left(\frac{v_{hj}}{1-v_{hj}}\right)}^{\lambda }.$
  2) When *q* = 2, the operator reduces to a Pythagorean fuzzy Dombi PHM (PFDPHM) operator:
    qROFDPHMa,b(Θ1,Θ2,…,Θn)=((1−(1/(1+(1d∑h=1d(2(a+b)|ph|(|ph|+1)∑i=1|Ph|∑j=i|Ph|1(af(uhi)+bf(uhj))))1λ)))12,(1/(1+(1d∑h=1d(2(a+b)|ph|(|ph|+1)∑i=1|Ph|∑j=i|Ph|1(ag(vhi)+bg(vhj))))1λ))12) (37)
    where $f(\mu_{hi})={\left(\frac{1-\mu_{{}_{hi}}^{2}}{\mu_{{}_{hi}}^{2}}\right)}^{\lambda },f(\mu_{hj})={\left(\frac{1-\mu_{{}_{hj}}^{2}}{\mu_{{}_{hj}}^{2}}\right)}^{\lambda },g(v_{hi})={\left(\frac{v_{{}_{hi}}^{2}}{1-v_{{}_{hi}}^{2}}\right)}^{\lambda }\;\mathrm{and}\;g(v_{hj})={\left(\frac{v_{{}_{hj}}^{2}}{1-v_{{}_{hj}}^{2}}\right)}^{\lambda }$.

### 3.2 *q*-Rung orthopair fuzzy Dombi weighted partitioned Heronian mean operators

The *q*ROFDPHM operator has the advantages of offering flexibility in describing fuzzy information, generating versatile operational rules for aggregating fuzzy information, and reflecting the interrelationships among different attributes. However, it does not consider the relative importance of attributes. To address this issue, weights are introduced, and a weighted *q*ROFDPHM operator is presented. The formal definition of this operator is as follows:

**Definition 12**. Let {Θ_1_, Θ_2_, …, Θ_*n*_} (where Θ_*i*_ = (*μ*_*i*_, *v*_*i*_) (*i* = 1, 2, …, *n*) be a collection of *q*ROFNs (*q* = 1, 2, …) that is partitioned into *d* distinct sorts *P*_*1*_,*P*_*2*_,*…*,*P*_*d*_, where *P*_*h*_ = {Θ_*h1*_, Θ_*h2*_,…, Θ_*h|Ph|*_ } (*h* = 1, 2, …, *d*) and |*P*_1_|+|*P*_2_|+…+|*P*_*d*_| = *n*, and let *w*_*i*_ denote the weight of Θ_*i*_, where *w*_*i*_ ∈ [0, 1] and *w*_1_ + *w*_2_ + … + *w*_*n*_ = 1. For any two real numbers *a* and *b* such that *a*, *b* ≥ *0* but *a* and *b* are not zero simultaneously, the *q*-rung orthopair fuzzy Dombi weighted partitioned Heronian mean (*q*ROFDWPHM) operator is defined as follows:

$$qROFDWPHM^{a,b}(\Theta_{1},\Theta_{2},\ldots ,\Theta_{n})=\frac{1}{d}{\left(\overset{d}{\underset{h=1}{⊕}}\left(\frac{2}{\left|P_{h}\right|(\left|P_{h}\right|+1)}\overset{\left|P_{h}\right|}{\underset{i=1}{⊕}}\overset{\left|P_{h}\right|}{\underset{j=i}{⊕}}{\left(w_{hi}\Theta_{hi}\right)}^{a}⊗{\left(w_{hj}\Theta_{hj}^{}\right)}^{b}\right)\right)}^{\frac{1}{a+b}}$$ (38)

**Theorem 5.** Let {Θ_1_, Θ_2_, …, Θ_*n*_} (where Θ_*i*_ = (*μ*_*i*_, *v*_*i*_) (*i* = 1, 2, …, *n*) be a collection of *q*ROFNs (*q* = 1, 2, …) that is partitioned into *d* distinct sorts *P*_*1*_,*P*_*2*_,*…*,*P*_*d*_, where *P*_*h*_ = {Θ_*h1*_, Θ_*h2*_, …, Θ_*h|Ph|*_ }, (*h* = 1, 2, …, *d*) and |*P*_1_|+|*P*_2_|+…+|*P*_*d*_| = *n*, let *a* and *b* be two real numbers such that *a*, *b* ≥ *0* but *a* and *b* are not zero simultaneously; let *λ* be a positive real number, and let *w*_*i*_ denote the weight of Θ_*i*_, where *w*_*i*_ ∈ [0, 1] and *w*_1_ + *w*_2_ … + *w*_*n*_ = 1. Then, the aggregated value produced by *q*ROFDWPHM is still a *q*ROFN and

qROFDWPHMa,b(Θ1,Θ2,…,Θn)=((1−(1/(1+(1d∑h=1d(2(a+b)|ph|(|ph|+1)∑i=1|Ph|∑j=i|Ph|1a/(whif(uhi))+b/(whjf(uhj))))1λ)))1q,(1/(1+(1d∑h=1d(2(a+b)|ph|(|ph|+1)∑i=1|Ph|∑j=i|Ph|1a/(whig(vhi))+b/(whjg(vhj))))1λ))1q) (39)

where $f(\mu_{hi})={\left(\frac{1-\mu_{{}_{hi}}^{q}}{\mu_{{}_{hi}}^{q}}\right)}^{\lambda },f(\mu_{hj})={\left(\frac{1-\mu_{{}_{hj}}^{q}}{\mu_{{}_{hj}}^{q}}\right)}^{\lambda },g(v_{hi})={\left(\frac{v_{{}_{hi}}^{q}}{1-v_{{}_{hi}}^{q}}\right)}^{\lambda },\;\mathrm{and}\;g(v_{hj})={\left(\frac{v_{{}_{hj}}^{q}}{1-v_{{}_{hj}}^{q}}\right)}^{\lambda }$.

The proof of this theorem is similar to the proof of Theorem 1; please refer to Appendix C. In addition, it is easy to prove that the *q*ROFDWPHM operator satisfies the properties of monotonicity and boundedness. The proofs of these facts are omitted.

### 3.3 *q*-Rung orthopair fuzzy Dombi power partitioned Heronian mean operators

In practice, during the MAGDM process, decision makers may assign some unreasonable evaluation values to the attributes. The negative effects of such values on the aggregation results can be reduced by incorporating the PA operator. Thus, a *q*-rung orthopair fuzzy Dombi power partitioned Heronian mean (*q*ROFDPPHM) operator and a *q*-rung orthopair fuzzy Dombi weighted power partitioned Heronian mean *(q*ROFDWPHM) operator are presented.

**Definition 13**. Let {Θ_1_, Θ_2_, …, Θ_*n*_} (where Θ_*i*_ = (*μ*_*i*_, *v*_*i*_) (*i* = 1, 2, …, *n*) be a collection of *q*ROFNs (*q* = 1, 2, …) that is partitioned into *d* distinct sorts *P*_*1*_,*P*_*2*_,*…*,*P*_*d*_, where *P*_*h*_ = {Θ_*h1*_, Θ_*h2*_,…, Θ_*h|Ph|*_} (*h* = 1, 2, …, *d*) and |*P*_1_|+|*P*_2_|+…+|*P*_*d*_| = *n*. For any two real numbers *a* and *b* such that *a*, *b* ≥ *0* but *a* and *b* are not zero simultaneously, the *q*-rung orthopair fuzzy Dombi power partitioned Heronian mean (*q*ROFDPPHM) operator is defined as follows:

$$\begin{array}{c} qROFDPPHM^{a,b}(\Theta_{1},\Theta_{2},\ldots ,\Theta_{n})=\\ \frac{1}{d}{\left(\overset{d}{\underset{h=1}{⊕}}\left(\frac{2}{\left|P_{h}\right|(\left|P_{h}\right|+1)}\overset{\left|P_{h}\right|}{\underset{i=1}{⊕}}\overset{\left|P_{h}\right|}{\underset{j=i}{⊕}}{\left(\frac{n(1+T(\Theta_{hi}))}{\sum_{k=1}^{n}\left(1+T(\Theta_{k})\right)}\Theta_{hi}\right)}^{a}⊗{\left(\frac{n(1+T(\Theta_{hj}))}{\sum_{k=1}^{n}\left(1+T(\Theta_{k})\right)}\Theta_{hj}\right)}^{b}\right)\right)}^{\frac{1}{a+b}} \end{array}$$ (40)

where $T(\Theta_{hi})=\sum_{j=1,j\neq i}^{n},Sup(\Theta_{hi},\Theta_{hj})$, *Sup*(Θ_*hi*_,Θ_*hj*_) = 1−*d*(Θ_*hi*_,Θ_*hj*_) is the Minkowski-type distance between Θ_hi_ and Θ_hj_ *Sup*(Θ_*i*_,Θ_*j*_) satisfies the following properties:

(1) *Sup*(Θ_*i*_,Θ_*j*_)∈[0,1];
(2) *Sup*(Θ_*i*_,Θ_*j*_) = *Sup*(Θ_*j*_,Θ_*i*_)
(3) *Sup*(Θ_*i*_,Θ_*j*_)> *Sup*(Θ_*h*_,Θ_*l*_), if *d*(Θ_*i*_,Θ_*j*_)< *d*(Θ_*h*_,Θ_*l*_).

To simplify Eq (40), let

$$w_{i}^{\prime }=\frac{1+T(\Theta_{i})}{\sum_{i=1}^{n}\left(1+T(\Theta_{i})\right)}$$ (41)

Then, *w*_*i*_’ ∈ [0, 1] and $\sum_{i=1}^{n}w_{i}^{\prime }=1$. Using this notation, Eq (40) can be expressed as:

$$qROFDPPHM^{a,b}(\Theta_{1},\Theta_{2},\ldots ,\Theta_{n})=\frac{1}{d}{\left(\overset{d}{\underset{h=1}{⊕}}\left(\frac{2}{\left|P_{h}\right|(\left|P_{h}\right|+1)}\overset{\left|P_{h}\right|}{\underset{i=1}{⊕}}\overset{\left|P_{h}\right|}{\underset{j=i}{⊕}}{\left(nw_{hi}^{\prime }\Theta_{hi}\right)}^{a}⊗{\left(nw_{hj}^{\prime }\Theta_{hj}\right)}^{b}\right)\right)}^{\frac{1}{a+b}}$$ (42)

**Theorem 6.** Let {Θ_1_, Θ_2_, …, Θ_*n*_} (where Θ_*i*_ = (*μ*_*i*_, *v*_*i*_) (*i* = 1, 2, …, *n*) be a collection of *q*ROFNs (*q* = 1, 2, …) that is partitioned into *d* distinct sorts *P*_*1*_,*P*_*2*_,*…*,*P*_*d*_, where *P*_*h*_ = {Θ_*h1*_, Θ_*h2*_,…, Θ_*h|Ph|*_} (*h* = 1, 2, …, *d*) and |*P*_1_|+|*P*_2_|+…+|*P*_*d*_| = *n*, let *a* and *b* be two real numbers such that *a*, *b* ≥ *0* but *a* and *b* are not zero simultaneously, and let *λ* be a positive real number. Then, the aggregated value produced by *q*ROFDPPHM is still a *q*ROFN and

qROFDPPHMa,b(Θ1,Θ2,…,Θn)=((1−(1/(1+(1d∑h=1d(2(a+b)|ph|(|ph|+1)∑i=1|Ph|∑j=i|Ph|1a/(nwhi′f(uhi))+b/(nwhj′f(uhj))))1λ)))1q,(1/(1+(1d∑h=1d(2(a+b)|ph|(|ph|+1)∑i=1|Ph|∑j=i|Ph|1a/(nwhi′g(vhi))+b/(nwhj′g(vhj))))1λ))1q) (43)

where

$$f(\mu_{hi})={\left(\frac{1-\mu_{{}_{hi}}^{q}}{\mu_{{}_{hi}}^{q}}\right)}^{\lambda },f(\mu_{hj})={\left(\frac{1-\mu_{{}_{hj}}^{q}}{\mu_{{}_{hj}}^{q}}\right)}^{\lambda },g(v_{hi})={\left(\frac{v_{{}_{hi}}^{q}}{1-v_{{}_{hi}}^{q}}\right)}^{\lambda },g(v_{hj})={\left(\frac{v_{{}_{hj}}^{q}}{1-v_{{}_{hj}}^{q}}\right)}^{\lambda }\;\mathrm{and}\;w_{i}^{\prime }=\frac{1+T(\Theta_{i})}{\sum_{k=1}^{n}\left(1+T(\Theta_{k})\right)}.$$

The proof of this theorem is similar to the proof of Theorem 5. It is omitted.

**Theorem 7 (Idempotency)**. Let {Θ_1_, Θ_2_, …, Θ_*n*_} (where Θ_*i*_ = (*μ*_*i*_, *v*_*i*_) (*i* = 1, 2, …, *n*) be a collection of *q*ROFNs (*q* = 1, 2, …), and let *a* and *b* be two real numbers such that *a*, *b* ≥ *0* but *a* and *b* are not zero simultaneously. If Θ_*i*_ = Θ = (*μ*, *v*) for all *i* = 1, 2, …, *n*, then

$$qROFDPPHM^{a,b}(\Theta_{1},\Theta_{2},\ldots ,\Theta_{m})=\Theta$$ (44)

**Theorem 8 (Monotonicity).** Let {Θ_1_, Θ_2_, …, Θ_*n*_} (where Θ_*i*_ = (*μ*_*i*_, *v*_*i*_) (*i* = 1, 2, …, *n*) and {Θ_1_’, Θ_2_’, …, Θ_*n*_*’*} (where Θ_*i*_*’* = (*μ*_*i*_*’*, *v*_*i*_*’*) (*i* = 1, 2, …, *n*) be two collections of *q*ROFNs (*q* = 1,2, …) ,,..), and let *a* and *b* be two real numbers such that *a*, *b* ≥ *0* but *a* and *b* are not zero simultaneously. If *μ*_*i*_ ≤ *μ*_*i*_*’* and *v*_*i*_ ≤ *v*_*i*_*’* for all *i* = 1, 2, …, *n*, then

$$qROFDPPHM^{a,b}(\Theta_{1},\Theta_{2},\ldots ,\Theta_{n})\leq qROFDPPHM^{a,b}(\Theta_{1}^{\prime },\Theta_{2}^{\prime },\ldots ,\Theta_{n}^{\prime })$$ (45)

**Theorem 9 (Boundedness)**. Let {Θ_1_, Θ_2_, …, Θ_*n*_} (where Θ_*i*_ = (*μ*_*i*_, *v*_*i*_) (*i* = 1, 2, …, *n*) be a collection of *q*ROFNs (*q* = 1, 2, …), let *a* and *b* be two real numbers such that *a*, *b* ≥ *0* but *a* and *b* are not zero simultaneously, and let Θ_*S*_ = (max(*μ*_*i*_), min(*v*_*i*_)) and Θ_*I*_ = (min(*μ*_*i*_), max(*v*_*i*_)). Then

$$\Theta_{I}\leq qROFDPPHM^{a,b}(\Theta_{1},\Theta_{2},\ldots ,\Theta_{n})\leq \Theta_{S}$$ (46)

The proofs of Theorem 7, Theorem 8 and Theorem 9 are similar to the proofs of Theorem 2, Theorem 3 and Theorem 4, respectively. They are omitted. The following are some special cases of the proposed *q*ROFDPPHM operator:

(1) Special cases with respect to parameters *a* and *b*.
  1) When *a* →0 or *b*→0 and *a* + *b*>0, then Eq (43) reduces to
    qROFDPPHMa,0(Θ1,Θ2,…,Θn)=qROFDPPHM0,b(Θ1,Θ2,…,Θn)=((1−(1/(1+(1d∑h=1d(2|ph|(|ph|+1)∑i=1|Ph|∑j=i|Ph|nwhi′f(uhi)))1λ)))1q,(1/(1+(1d∑h=1d(2|ph|(|ph|+1)∑i=1|Ph|∑j=i|Ph|nwhi′g(vhi)))1λ))1q). (47)
    which is a *q*-rung orthopair fuzzy Dombi partitioned power generalized heavy averaging operator.
  2) When *b*→0 and all the *q*ROFNs are partitioned into one sort, then Eq (43) reduces to
    qROFDPPHMa,0(Θ1,Θ2,…,Θn)=((1−(1/(1+(2n(n+1)∑i=1n∑j=innwhi′f(uhi))1λ)))1q,(1/(1+(2n(n+1)∑i=1n∑j=innwhi′g(vhi))1λ))1q). (48)
  3) When *b*→0 and all the *q*ROFNs are partitioned into n sorts, then Eq (43) reduces to
    $$\begin{array}{l} qROFDPPHM^{a,0.}(\Theta_{1},\Theta_{2},\ldots ,\Theta_{n})=\\ \left({\left(1-\left(1/\left(1+{\left(\frac{1}{n}\sum_{h=1}^{n}\left(nw_{h}^{'}f\left(u_{h}\right)\right)\right)}^{\frac{1}{\lambda }}\right)\right)\right)}^{\frac{1}{q}},{\left(1/\left(1+{\left(\frac{1}{n}\sum_{h=1}^{n}\left(nw_{h}^{'}g\left(v_{h}\right)\right)\right)}^{\frac{1}{\lambda }}\right)\right)}^{\frac{1}{q}}\right). \end{array}$$ (49)
  4) When *a*→1 and *b*→1, then Eq (43) reduces to
    qROFDPPHM1,1(Θ1,Θ2,…,Θn)=((1−(1/(1+(1d∑h=1d(4|ph|(|ph|+1)∑i=1|Ph|∑j=i|Ph|nwhi′f(uhi)whj′f(uhj)whi′f(uhi)+whj′f(uhj)))1λ)))1q,(1/(1+(1d∑h=1d(4|ph|(|ph|+1)∑i=1|Ph|∑j=i|Ph|nwhi′g(uhi)whj′g(uhj)whi′g(uhi)+whj′g(uhj)))1λ))1q). (50)
(2) Special cases with respect to parameter *q*.
  1) When *q* = 1, the *q*ROFDPPHM operator reduces to an intuitionistic fuzzy Dombi power PHM (IFDPPHM) operator:
    qROFDPPHMa,b(Θ1,Θ2,…,Θn)=((1−(1/(1+(1d∑h=1d(2(a+b)|ph|(|ph|+1)∑i=1|Ph|∑j=i|Ph|1a/(nwhi′f(uhi))+b/(nwhj′f(uhj))))1λ))),(1/(1+(1d∑h=1d(2(a+b)|ph|(|ph|+1)∑i=1|Ph|∑j=i|Ph|1a/(nwhi′g(vhi))+b/(nwhj′g(vhj))))1λ))) (51)
    where
    $$f(\mu_{hi})={\left(\frac{1-\mu_{{}_{hi}}^{}}{\mu_{{}_{hi}}^{}}\right)}^{\lambda },f(\mu_{hj})={\left(\frac{1-\mu_{{}_{hj}}^{}}{\mu_{{}_{hj}}^{}}\right)}^{\lambda },g(v_{hi})={\left(\frac{v_{{}_{hi}}^{}}{1-v_{{}_{hi}}^{}}\right)}^{\lambda },g(v_{hj})={\left(\frac{v_{{}_{hj}}^{}}{1-v_{{}_{hj}}^{}}\right)}^{\lambda }\;\mathrm{and}\;w_{i}^{\prime }=\frac{1+T(\Theta_{i})}{\sum_{k=1}^{n}\left(1+T(\Theta_{k})\right)}.$$
  2) When *q* = 2, the *q*ROFDPPHM operator reduces to a Pythagorean fuzzy Dombi power PHM (PFDPPHM) operator:
    qROFDPPHMa,b(Θ1,Θ2,…,Θn)=((1−(1/(1+(1d∑h=1d(2(a+b)|ph|(|ph|+1)∑i=1|Ph|∑j=i|Ph|1a/(nwhi′f(uhi))+b/(nwhj′f(uhj))))1λ)))12,(1/(1+(1d∑h=1d(2(a+b)|ph|(|ph|+1)∑i=1|Ph|∑j=i|Ph|1a/(nwhi′g(vhi))+b/(nwhj′g(vhj))))1λ))12) (52)
    where
    $$f(\mu_{hi})={\left(\frac{1-\mu_{{}_{hi}}^{2}}{\mu_{{}_{hi}}^{2}}\right)}^{\lambda },f(\mu_{hj})={\left(\frac{1-\mu_{{}_{hj}}^{2}}{\mu_{{}_{hj}}^{2}}\right)}^{\lambda },g(v_{hi})={\left(\frac{v_{{}_{hi}}^{2}}{1-v_{{}_{hi}}^{2}}\right)}^{\lambda },g(v_{hj})={\left(\frac{v_{{}_{hj}}^{2}}{1-v_{{}_{hj}}^{2}}\right)}^{\lambda }\;\mathrm{and}\;w_{i}^{\prime }=\frac{1+T(\Theta_{i})}{\sum_{k=1}^{n}\left(1+T(\Theta_{k})\right)}.$$

### 3.4 *q*-Rung orthopair fuzzy Dombi weighted power partitioned Heronian mean operators

In this section, weights are introduced to capture the relative importance of attributes, and the weighted form of the *q*ROFDPPHM operator is proposed.

**Definition 14**. Let {Θ_1_, Θ_2_, …, Θ_*n*_} (where Θ_*i*_ = (*μ*_*i*_, *v*_*i*_) (*i* = 1, 2, …, *n*) be a collection of *q*ROFNs (*q* = 1, 2, …) that is partitioned into *d* distinct sorts *P*_*1*_,*P*_*2*_,*…*,*P*_*d*_, where *P*_*h*_ = {Θ_*h1*_, Θ_*h2*_,…, Θ_*h|Ph|*_} (*h* = 1, 2, …, *d*) and |*P*_1_|+|*P*_2_|+…+|*P*_*d*_| = *n*, and let *w*_*i*_ denote the weight of Θ_*i*_, where *w*_*i*_ ∈ [0, 1] and *w*_1_ + *w*_2_ + … + *w*_*n*_ = 1. For any two real numbers *a* and *b* such that *a*, *b* ≥ *0* but *a* and *b* are not zero simultaneously, the *q*-rung orthopair fuzzy Dombi weighted partitioned Heronian mean (*q*ROFDWPHM) operator is defined as follows:

$$\begin{array}{c} qROFDWPPHM^{a,b}(\Theta_{1},\Theta_{2},\ldots ,\Theta_{n})=\\ \frac{1}{d}{\left(\overset{d}{\underset{h=1}{⊕}}\left(\frac{2}{\left|P_{h}\right|(\left|P_{h}\right|+1)}\overset{\left|P_{h}\right|}{\underset{i=1}{⊕}}\overset{\left|P_{h}\right|}{\underset{j=i}{⊕}}{\left(\frac{nw_{hi}(1+T(\Theta_{hi}))}{\sum_{k=1}^{n}w_{k}(1+T(\Theta_{k}))}\Theta_{hi}\right)}^{a}⊗{\left(\frac{nw_{hj}(1+T(\Theta_{hj}))}{\sum_{k=1}^{n}w_{k}(1+T(\Theta_{k}))}\Theta_{hj}\right)}^{b}\right)\right)}^{\frac{1}{a+b}} \end{array}$$ (53)

where $T(\Theta_{hi})=\sum_{j=1,j\neq i}^{n},Sup(\Theta_{hi},\Theta_{hj})$, *Sup*(Θ_*hi*_,Θ_*hj*_) = 1−*d*(Θ_*hi*_,Θ_*hj*_) and *d*(Θ_*hi*_,Θ_*hj*_) is the Minkowski-type distance between Θ_hi_ and Θ_hj_ *Sup*(Θ_*i*_,Θ_*j*_) has the following properties:

(1) *Sup*(Θ_*i*_,Θ_*j*_)∈[0,1];
(2) *Sup*(Θ_*i*_,Θ_*j*_) = *Sup*(Θ_*j*_,Θ_*i*_)
(3) *Sup*(Θ_*i*_,Θ_*j*_)> *Sup*(Θ_*h*_,Θ_*l*_), if *d*(Θ_*i*_,Θ_*j*_)< *d*(Θ_*h*_,Θ_*l*_).

To simplify Eq (53), let

$$w_{i}^{\prime }=\frac{1+T(\Theta_{i})}{\sum_{i=1}^{n}\left(1+T(\Theta_{i})\right)}$$ (54)

Then, *w*_*i*_’ ∈ [0, 1] and $\sum_{i=1}^{n}w_{i}^{\prime }=1$. Using this notation, Eq (53) can be expressed as:

$$\begin{array}{c} qROFDWPPHM^{a,b}(\Theta_{1},\Theta_{2},\ldots ,\Theta_{n})=\\ \frac{1}{d}{\left(\overset{d}{\underset{h=1}{⊕}}\left(\frac{2}{\left|P_{h}\right|(\left|P_{h}\right|+1)}\overset{\left|P_{h}\right|}{\underset{i=1}{⊕}}\overset{\left|P_{h}\right|}{\underset{j=i}{⊕}}{\left(\frac{nw_{hi}^{\prime }w_{hi}}{\sum_{k=1}^{n}w_{k}w_{k}^{\prime }}\Theta_{hi}\right)}^{a}⊗{\left(\frac{nw_{hj}^{\prime }w_{hj}}{\sum_{k=1}^{n}w_{k}w_{k}^{\prime }}\Theta_{hj}\right)}^{b}\right)\right)}^{\frac{1}{a+b}} \end{array}$$ (55)

**Theorem 10.** Let {Θ_1_, Θ_2_, …, Θ_*n*_} (where Θ_*i*_ = (*μ*_*i*_, *v*_*i*_) (*i* = 1, 2, …, *n*) be a collection of *q*ROFNs (*q* = 1, 2, …) that is partitioned into *d* distinct sorts *P*_*1*_,*P*_*2*_,*…*,*P*_*d*_, where *P*_*h*_ = {Θ_*h1*_, Θ_*h2*_,…, Θ_*h|Ph|*_} (*h* = 1, 2, …, *d*) and |*P*_1_|+|*P*_2_|+…+|*P*_*d*_| = *n*. Let *w*_*i*_ denote the weight of Θ_*i*_, where *w*_*i*_ ∈ [0, 1] and *w*_1_ + *w*_2_ + … + *w*_*n*_ = 1, let *a* and *b* be two real numbers such that *a*, *b* ≥ *0* but *a* and *b* are not zero simultaneously, and let *λ* be a positive real number. Then, the aggregated value produced by *q*ROFDPPHM is still a *q*ROFN and

qROFDWPPHMa,b(Θ1,Θ2,…,Θn)=((1−(1/(1+(1d∑h=1d(2(a+b)|ph|(|ph|+1)∑i=1|Ph|∑j=i|Ph|1/(a/(nwhi′whi∑k=1nwk′wkf(uhi))+b/(nwhj′whj∑k=1nwk′wkf(uhj)))1λ))))1q,(1/(1+(1d∑h=1d(2(a+b)|ph|(|ph|+1)∑i=1|Ph|∑j=i|Ph|1/(a/(nwhi′whi∑k=1nwk′wkg(vhi))+b/(nwhj′whj∑k=1nwk′wkg(vhj))))1λ))1q) (56)

where

$$f(\mu_{hi})={\left(\frac{1-\mu_{{}_{hi}}^{q}}{\mu_{{}_{hi}}^{q}}\right)}^{\lambda },f(\mu_{hj})={\left(\frac{1-\mu_{{}_{hj}}^{q}}{\mu_{{}_{hj}}^{q}}\right)}^{\lambda },g(v_{hi})={\left(\frac{v_{{}_{hi}}^{q}}{1-v_{{}_{hi}}^{q}}\right)}^{\lambda },g(v_{hj})={\left(\frac{v_{{}_{hj}}^{q}}{1-v_{{}_{hj}}^{q}}\right)}^{\lambda }\;\mathrm{and}\;w_{i}^{\prime }=\frac{1+T(\Theta_{i})}{\sum_{k=1}^{n}\left(1+T(\Theta_{k})\right)}.$$

The proof of the above theorem is similar to the proof of Theorem 5. It is omitted.

## 4. Novel MAGDM method based on the presented operator

In this section, a novel MAGDM method is proposed based on the presented *q*ROFDWPPHM operator.

A MAGDM problem based on *q*ROFNs can be described through a set of alternatives *A* = {*A*_1_, *A*_2_, …, *A*_m_}, a set of attributes *C* = {*C*_1_, *C*_2_, …, *C*_n_}, a set of weights *w* = {*w*_1_, *w*_2_, …, *w*_n_} (where *w*_i_ ∈ [0,1] and *w*_1_ + *w*_2_ + … + *w*_*n*_ = 1), and a group of decision makers *D* = {*D*_1_, *D*_2_, …, *D*_*t*_} whose weight vector is ω = {ω_1_, ω_2_, …, ω_*t*_} (where *ω*_*i*_ ∈ [0,1] (*i* = 1, 2, …, *t*) and *ω*_1_ + *ω*_2_+ … + *ω*_*t*_ = 1). Suppose that these n attributes (*C*_1_, *C*_2_, …, *C*_n_) are divided into *d* different classes *P*_1_, *P*_2_, *…*, *P*_*d*_, that there is at least one and at most n attributes in each class, and that all the attributes in each class are related to each other, while the attributes in different classes are not related. The problem is always coupled with a *q*-rung orthopair fuzzy decision matrix *M*_*k*_ = ${\left[\Theta_{ij}^{k}\right]}_{m,n}$, where *i* = 1, 2, …, *m*, *j =* 1, 2,*…*,*n* and $\Theta_{ij}^{k}=(\mu_{ij}^{k},v_{ij}^{k})$ (*k =* 1, 2,*…*,*t*) is a *q*ROFN that stands for the evaluation value of alternative *A*_*i*_ with respect to attribute *C*_*j*_ given by decision maker *D*_*k*_.

On the basis of the components above, the problem can be described as follows: Make a decision with the help of a ranking of the elements of *A* based on *M*_*k*_, *w* and *ω*. Using the *q*ROFDWPPHM operator, the problem is solved according to the following steps:

(1) Normalize the decision matrix. In real decision making, the attributes in each MAGDM problem are divided into two types, i.e., cost attributes and benefit attributes, which have positive and negative effects, respectively, on the aggregation results. To eliminate this difference in attribute types, it is necessary to convert the attributes to the same type. The following equation provides the rules for such conversion:
  Mk'={[μijk,vijk]m,n,ifCjisabenefitattribute[vijk,μijk]m,n,ifCjisacostattribute (57)
(2) Incorporate the evaluation information of the decision makers into the collective information. Taking the normalized decision matrix *M*_*k*_’ and the weight set *ω* as input, the collective information of each alternative can be computed by the *q*ROFDWPPHM operator as follows:
  $$\Theta_{ij}^{}=qROFDWPPHM^{a,b}(\Theta_{ij}^{1},\Theta_{ij}^{2},\ldots ,\Theta_{ij}^{t})$$ (58)
(3) Incorporate the evaluation information of each attribute into the comprehensive evaluation value of each alternative. Taking each of the columns of the collective information decision matrix and the weight set as input, the collective information of each alternative can be computed by the proposed *q*ROFDWPPHM operator, which is shown as follows:
  $$\Theta_{i}^{}=qROFDWPPHM^{a,b}(\Theta_{i1}^{},\Theta_{i2}^{},\ldots ,\Theta_{in}^{})$$ (59)
(4) In accordance with Eqs (3) and (4), calculate the score and accuracy of the comprehensive evaluation value of each alternative.
(5) Rank all the alternatives and select a proper alternative. In accordance with the comparison rules in Definition 4, a ranking of the alternatives is generated. With the help of the generated ranking, an appropriate alternative can be selected by the decision maker.

## 5. Example, experiments and comparisons

In this section, the process of the proposed MAGDM method is first illustrated via a practical example. Then, a set of experiments is carried out to explore the influence of different parameter values on the aggregation results. Finally, the validity of the method is verified by comparisons with the existing MAGDM methods.

### 5.1 Example

A MAGDM problem about company location selection [8] is provided to illustrate the proposed approach. In this example, an investment enterprise wants to invest some money into a company. There are five possible companies, *A*_1_, *A*_2_, *A*_3_, *A*_4_, and *A*_5_. To make a proper decision, the investment enterprise invites three experts *D*_1_, *D*_2_, and *D*_3_ to evaluate the alternatives with respect to four attributes *C*_1_, *C*_2_, *C*_3,_ and *C*_4_, where *C*_1_ denotes the risk analysis, *C*_2_ denotes the growth analysis, *C*_3_ denotes the social-political impact analysis, and *C*_4_ denotes the environmental impact analysis. The relative importance of the four attributes and the three decision makers is measured by the weights in *w* = {0.2,0.1,0.3,0.4} and *ω* = {0.35,0.40,0.25}, respectively. The decision matrices of the attributes of the five companies provided by the decision makers *D*_1_, *D*_2_, and *D*_3_ are shown in Tables 1–3. To make a reasonable decision, the interrelationships among attributes should be considered. Therefore, assume that the attributes are divided into two classes, *P*_1_ = {*C*_1_, *C*_2_} and *P*_2_ = {*C*_3,_ *C*_4_}, and that there are interrelationships between the two attributes in each class, whereas the attributes in *P*_1_ are not related to those in *P*_2_.

**Table 1:** The *q*-rung orthopair fuzzy decision matrix M_1_ given by D_1_.

	*C*_*1*_	*C*_*2*_	*C*_*3*_	*C*_*4*_
*A*_1_	(0.5,0.4)	(0.5,0.4)	(0.2,0.6)	(0.4,0.4)
*A*_2_	(0.7,0.3)	(0.7,0.3)	(0.6,0.2)	(0.6,0.2)
*A*_3_	(0.5,0.4)	(0.6,0.4)	(0.6,0.2)	(0.5,0.3)
*A*_4_	(0.8,0.2)	(0.7,0.2)	(0.4,0.2)	(0.5,0.2)
*A*_5_	(0.4,0.3)	(0.4,0.2)	(0.4,0.5)	(0.4,0.6)

**Table 2:** The *q*-rung orthopair fuzzy decision matrix M_2_ given by D_2_.

	*C*_*1*_	*C*_*2*_	*C*_*3*_	*C*_*4*_
*A*_*1*_	(0.4,0.5)	(0.6,0.2)	(0.5,0.4)	(0.5,0.3)
*A*_*2*_	(0.5,0.4)	(0.6,0.2)	(0.6,0.3)	(0.7,0.3)
*A*_*3*_	(0.4,0.5)	(0.3,0.5)	(0.4,0.4)	(0.2,0.6)
*A*_*4*_	(0.5,0.4)	(0.7,0.2)	(0.4,0.4)	(0.6,0.2)
*A*_*5*_	(0.6,0.3)	(0.7,0.2)	(0.4,0.2)	(0.7,0.2)

**Table 3:** The *q*-rung orthopair fuzzy decision matrix M_3_ given by D_3_.

	*C*_*1*_	*C*_*2*_	*C*_*3*_	*C*_*4*_
*A*_*1*_	(0.4,0.2)	(0.5,0.2)	(0.5,0.3)	(0.5,0.2)
*A*_*2*_	(0.5,0.3)	(0.5,0.3)	(0.6,0.2)	(0.7,0.2)
*A*_*3*_	(0.4,0.4)	(0.3,0.4)	(0.4,0.3)	(0.3,0.3)
*A*_*4*_	(0.5,0.3)	(0.5,0.3)	(0.3,0.5)	(0.5,0.2)
*A*_*5*_	(0.6,0.2)	(0.6,0.4)	(0.4,0.4)	(0.6,0.3)

In the following, the proposed method is used to solve the MAGDM problem. The selection process consists of the following five steps:

(1) Normalize the decision matrix. Since all attributes are benefit attributes, this step is skipped. The normalized decision matrix *M*_*k*_’ is equal to *M*_*k*_, i.e., *M*_*k*_’ = *M*_*k*_.
(2) Incorporate the evaluation information of the decision makers into the collective information. Using Eq (57) and taking the normalized decision matrix *M*_*k*_’ and the weight set *ω* as input, the evaluation information of the three decision makers is aggregated into collective information by the proposed *q*ROFDWPPHM operator (let the values of the parameters be *a* = 1, *b* = 2 and *λ* = 1.5, and let the decision matrices be divided into three classes *P*^*’*^_1_ = {*M*_1_}, *P*^*’*^_2_ = {*M*_2_} and *P*^*’*^_3_ = {*M*_3_}). The collective decision matrix is presented in Table 4.

**Table 4:** Collective *q*-rung orthopair fuzzy decision matrix.

	*C*_*1*_	*C*_*2*_	*C*_*3*_	*C*_*4*_
*A*_*1*_	(0.8039,0.2729)	(0.7619,0.4302)	(0.9168,0.1879)	(0.8252,0.3258)
*A*_*2*_	(0.6879,0.3648)	(0.6453,0.4277)	(0.7007,0.4844)	(0.6770,0.4845)
*A*_*3*_	(0.8028,0.2720)	(0.8193,0.2735)	(0.7701,0.4222)	(0.8784,0.2661)
*A*_*4*_	(0.6533,0.4221)	(0.6130,0.5258)	(0.8414,0.4073)	(0.7629,0.5325)
*A*_*5*_	(0.8155,0.4044)	(0.8109,0.5179)	(0.8400,0.2570)	(0.8073,0.1969)

(3) Incorporate the evaluation information of each attribute into the comprehensive evaluation value of each alternative. Using Eq (58) and taking each of the columns of the collective information decision matrix and the weight set *w* as input, the evaluation information of the attributes is aggregated into a comprehensive evaluation value by the proposed *q*ROFDWPPHM operator. The comprehensive evaluation value is presented as follows:
  $$\begin{array}{l} \Theta_{1}=(0.1615,0.7071),\Theta_{2}=(0.3150,0.5448),\Theta_{3}=(0.1731,0.6858)\\ \Theta_{4}=(0,2683,0.5262),\Theta_{5}=(0.1779,0.6704), \end{array}$$
(4) Calculate the score and accuracy of the comprehensive evaluation value of each alternative. In accordance with Eqs (3) and (4), the score and accuracy of the comprehensive evaluation value of each company is computed. The results are shown in Table 5.

**Table 5:** The calculated scores and accuracies.

Indicator	*A*_*1*_	*A*_*2*_	*A*_*3*_	*A*_*4*_	*A*_*5*_
**Score**	-0.5456	-0.2298	-0.5127	-0.2579	-0.4925
**Accuracy**	0.8686	0.8598	0.8989	0.7945	0.8483

(5) Rank all the alternatives and select a proper alternative. On the basis of the calculated results in Table 5, a ranking of the five companies is obtained in accordance with Definition 4:
  $$A_{2}>A_{4}>A_{5}>A_{3}>A_{1}$$

Based on this ranking, company A_2_ will probably be selected by the investment enterprise.

### 5.2 Experiments

In the following, the effects of assigning different values to parameters on the ranking results in the example are explored.

(1) Experiment 1 was carried out to show the effect of assigning different values to the parameter *q* on the ranking results. The results of the experiment are the scores and rankings of the five alternatives, which are shown in Table 6 (suppose *a* = 1, *b* = 2, *λ* = 1.5 and *p* = 3). From the table, it can be found that the ranking will change as the value of the parameter *q* changes. When *q* = 2, the ranking is *A*_2_ *> A*_4_ *> A*_5_ *> A*_3_ *> A*_1_. When *q* = 3,4,5,6,7, the rankings are all *A*_2_ *> A*_4_ *> A*_5_ *> A*_1_ *> A*_3_. When *q* = 8, the ranking is *A*_2_ *> A*_4_ *> A*_1_ *> A*_5_ *> A*_3_. Although the rankings have changed, the first and second alternatives remain the same. The assignment of a reasonable value for *q* depends on the values of the attributes because these attribute values must satisfy the condition that 0 ≤ *v*^*q*^ + *μ*^*q*^ ≤ 1. From Table 4, the values for each criterion do not satisfy *v* + *μ* ≤ 1 but do satisfy *v*^*2*^ + *μ*^*2*^ ≤ 1; thus, in this example, *q* should be assigned a value of at least 2.

**Table 6:** The results of experiment 1.

*q*	Scores of the five alternatives	Ranking
*q* = 2	S_1_ = -0.3465, S_2_ = -0.0490, S_3_ = -0.3294, S_4_ = -0.0642, S_5_ = -0.2566	*A*_2_>*A*_4_>*A*_5_>*A*_3_>*A*_1_
*q* = 3	S_1_ = = -0.1631, S_2_ = 0.0198, S_3_ = -0.1664, S_4_ = 0.0082, S_5_ = -0.0886	*A*_2_>*A*_4_>*A*_5_>*A*_1_>*A*_3_
*q* = 4	S_1_ = = -0.0654, S_2_ = 0.0269, S_3_ = -0.0764, S_4_ = 0.0179, S_5_ = -0.0270	*A*_2_>*A*_4_>*A*_5_>*A*_1_>*A*_3_
*q* = 5	S_1_ = -0.0228, S_2_ = 0.0200, S_3_ = -0.0335, S_4_ = 0.0134, S_5_ = -0.0083	*A*_2_>*A*_4_>*A*_5_>*A*_1_>*A*_3_
*q* = 6	S_1_ = -0.0067, S_2_ = 0.0131, S_3_ = -0.0139, S_4_ = 0.0086, S_5_ = -0.0029	*A*_2_>*A*_4_>*A*_5_>*A*_1_>*A*_3_
*q* = 7	S_1_ = -0.0027, S_2_ = 0.0085, S_3_ = -0.0052, S_4_ = 0.0046, S_5_ = -0.0008	*A*_2_>*A*_4_>*A*_5_>*A*_1_>*A*_3_
*q* = 8	S_1_ = 0.0005, S_2_ = 0.0048, S_3_ = -0.0018, S_4_ = 0.0025, S_5_ = 0.0000	*A*_2_>*A*_4_>*A*_1_>*A*_5_>*A*_3_

(2) Experiment 2 was carried out to show the effect of assigning different values to the parameter *p* (*p*>1) on the ranking results. The results of the experiment are the scores and rankings of the five alternatives, which are shown in Table 7 (suppose a = 1, b = 2, *λ* = 1.5 and *q* = 2). From the table, it can be found that the rankings and the values of the score function remain almost the same for different values of the parameter *p*, which indicates that using different values for *p* has no obvious influence on the ranking results in this example.

**Table 7:** The results of experiment 2.

*p*	Scores of the five alternatives	Ranking
*p* = 1.1	S_1_ = -0.3458, S_2_ = -0.0492, S_3_ = -0.3284, S_4_ = -0.0638, S_5_ = -0.2564	*A*_2_>*A*_4_>*A*_5_>*A*_3_>*A*_1_
*p* = 1.5	S_1_ = -0.3461, S_2_ = -0.0491, S_3 = -_0.3287, S_4_ = -0.0640, S_5_ = -0.2564	*A*_2_>*A*_4_>*A*_5_>*A*_3_>*A*_1_
*p* = 2	S_1_ = -0.3462, S_2_ = -0.0491, S_3_ = -0.3291, S_4_ = 0.0640, S_5_ = -0.2564	*A*_2_>*A*_4_>*A*_5_>*A*_3_>*A*_1_
*p* = 3	S_1_ = -0.3465, S_2_ = -0.0490, S_3_ = -0.3294, S_4_ = -0.0642, S_5_ = -0.2566	*A*_2_>*A*_4_>*A*_5_>*A*_3_>*A*_1_
*p* = 5	S_1_ = -0.3465, S_2_ = -0.4900, S_3_ = -0.3295, S_4_ = -0.0642, S_5_ = -0.2565	*A*_2_>*A*_4_>*A*_5_>*A*_3_>*A*_1_
*p* = 10	S_1_ = -0.3466, S_2_ = -0.4900, S_3_ = -0.3298, S_4_ = -0.0642, S_5_ = -0.2563	*A*_2_>*A*_4_>*A*_5_>*A*_3_>*A*_1_
*p* = 50	S_1_ = -0.3467, S_2_ = -0.4900, S_3_ = -0.3300, S_4_ = -0.0644, S_5_ = -0.2563	*A*_2_>*A*_4_>*A*_5_>*A*_3_>*A*_1_
*p* = 100	S_1_ = -0.3467, S_2_ = -0.4900, S_3_ = -0.3301, S_4_ = -0.0645, S_5_ = -0.2564	*A*_2_>*A*_4_>*A*_5_>*A*_3_>*A*_1_

(3) Experiment 3 was carried out to show the effect of assigning different values to parameters *a* and *b* on the ranking results. The results of the experiment are the scores and rankings of the five alternatives, which are shown in Table 8 (suppose *λ* = 1.5, *q* = 2, *p* = 3). It can be seen from the table that which alternative is best depends on the sum of a and b. When the sum of a and b is less than 4, the best alternative is always A_2,_ but when the sum of a and b is greater than 4, the best alternative becomes A_4_. When the sum of a and b equals 4, the best alternative depends on the value of b. When *b* > 1.8, the best alternative changes from *A*_2_ to *A*_4,_ and the order of the other choices remains the same. As the parameters *a* and *b* increase, the interrelation among attribute values becomes stronger and stronger. Thus, the interaction strength significantly affects the ranking results. In practice, the risk degree of decision makers can be expressed by assigning reasonable parameters *a* and *b*. The greater the parameter is, the greater the risk. For example, in this case, if a decision maker prefers the fourth alternative, a larger value can be assigned to b. Otherwise, smaller values are specified for a and b (to ensure their sum at most 4).

**Table 8:** The results of experiment 3.

*a and b*	Scores of the five alternatives	Ranking
*a = 0*, *b = 1*	S_1_ = -0.1407, S_2_ = 0.1217, S_3_ = -0.2255, S_4_ = 0.0666, S_5_ = -0.1605	*A*_2_>*A*_4_>*A*_1_>*A*_5_>*A*_3_
*a = 1*, *b = 0*	S_1_ = -0.2142, S_2_ = 0.1034, S_3_ = -0.1507, S_4_ = 0.0134, S_5_ = -0.1535	*A*_2_>*A*_4_>*A*_3_>*A*_5_>*A*_1_
*a = 1*, *b = 1*	S_1_ = -0.2888, S_2_ = 0.0172, S_3_ = -0.2718, S_4_ = -0.0286, S_5_ = -0.2177	*A*_2_>*A*_4_>*A*_5_>*A*_3_>*A*_1_
*a = 3*, *b = 1*	S_1_ = -0.4097, S_2_ = -0.1024, S_3_ = -0.3451, S_4_ = -0.1048, S_5_ = -0.2877	*A*_2_>*A*_4_>*A*_5_>*A*_3_>*A*_1_
*a = 2*.*2*, *b = 1*.*8*	S_1_ = -0.4050, S_2_ = -0.1028, S_3_ = -0.3602, S_4_ = -0.1029, S_5_ = -0.2874	*A*_2_>*A*_4_>*A*_5_>*A*_3_>*A*_1_
*a = 2*, *b = 1*.*9*	S_1_ = -0.3997, S_2_ = -0.0979, S_3_ = -0.3585, S_4_ = -0.0993, S_5_ = -0.2846	*A*_2_>*A*_4_>*A*_5_>*A*_3_>*A*_1_
*a = 2*.*1*, *b = 1*.*9*	S_1_ = -0.4042, S_2_ = -0.1028, S_3_ = -0.3616, S_4_ = -0.1026, S_5_ = -0.2876	*A*_4_>*A*_2_>*A*_5_>*A*_3_>*A*_1_
*a = 2*, *b = 2*	S_1_ = -0.4034, S_2_ = -0.1028, S_3_ = -0.3631, S_4_ = -0.1022, S_5_ = -0.2875	*A*_4_>*A*_2_>*A*_5_>*A*_3_>*A*_1_
*a = 1*, *b = 3*	S_1_ = -0.3923, S_2_ = -0.1022, S_3_ = -0.3739, S_4_ = -0.0954, S_5_ = -0.2863	*A*_4_>*A*_2_>*A*_5_>*A*_3_>*A*_1_
*a = 3*, *b = 3*	S_1_ = -0.4760, S_2_ = -0.1849, S_3_ = -0.4296, S_4_ = -0.1558, S_5_ = -0.3367	*A*_4_>*A*_2_>*A*_5_>*A*_3_>*A*_1_
*a = 1*, *b = 5*	S_1_ = -0.4628, S_2_ = -0.1858, S_3_ = -0.4431, S_4_ = -0.1490, S_5_ = -0.3339	*A*_4_>*A*_2_>*A*_5_>*A*_3_>*A*_1_
*a = 5*, *b = 1*	S_1_ = -0.4820, S_2_ = -0.1823, S_3_ = -0.4037, S_4_ = -0.1560, S_5_ = -0.3381	*A*_4_>*A*_2_>*A*_5_>*A*_3_>*A*_1_
*a = 3*, *b = 5*	S_1_ = -0.5241, S_2_ = -0.2501, S_3_ = -0.4874, S_4_ = -0.1975, S_5_ = -0.3747	*A*_4_>*A*_2_>*A*_5_>*A*_3_>*A*_1_
*a = 5*, *b = 5*	S_1_ = -0.5658, S_2_ = -0.3020, S_3_ = -0.5235, S_4_ = -0.2331, S_5_ = -0.4085	*A*_4_>*A*_2_>*A*_5_>*A*_3_>*A*_1_

(4) Experiment 4 was carried out to show the effect of assigning different values to parameter *λ* on the ranking results. The results of the experiment are the scores and rankings of the five alternatives, which are shown in Table 9 (suppose *a* = 1, *b* = 2, *q* = 2 and *p* = 3). As seen from the table, the top-ranking alternative is *A*_4_ when *λ* < 1, the top-ranking alternative becomes *A*_2_ when *λ* ≥ 1, and the scores of *A*_1_, *A*_2_, *A*_3_, *A*_4_ and *A*_5_ gradually increase as *λ* increases. This indicates that the parameter *λ* can be regarded as the “decision maker’s attitude”. The smaller the value of the parameter *λ* is, the more pessimistic the decision maker, and vice versa. When the decision maker’s attitude is pessimistic to a certain extent (*λ* < 1), the best alternative will change, that is, the original first-place attribute will drop to second place. We can divide the attitudes of the three decision makers according to the following rankings and the range of their corresponding parameter *λ*: pessimistic (0 < *λ* ≤ 1), neutral (1< *λ* ≤5) and optimistic (*λ>*5).

**Table 9:** The results of experiment 4.

*λ*	Scores of the five alternatives	Ranking
*λ* = 0.5	S_1_ = -0.8925, S_2_ = -0.7335, S_3_ = -0.8641, S_4_ = -0.7057, S_5_ = -0.8707	*A*_4_>*A*_2_>*A*_3_>*A*_5_>*A*_1_
*λ* = 0.9	S_1_ = -0.5907, S_2_ = -0.2855, S_3_ = -0.5427, S_4_ = -0.2739, S_5_ = -0.5233	*A*_4_>*A*_2_>*A*_5_>*A*_3_>*A*_1_
*λ* = 1	S_1_ = -0.5334, S_2_ = -0.2247, S_3_ = -0.4893, S_4_ = -0.2196, S_5_ = -0.4591	*A*_4_>*A*_2_>*A*_5_>*A*_3_>*A*_1_
*λ* = 2	S_1_ = -0.2467, S_2_ = -0.2467, S_3_ = -0.2522, S_4_ = 0.0130, S_5_ = -0.1594	*A*_2_>*A*_4_>*A*_5_>*A*_1_>*A*_3_
*λ* = 3	S_1_ = -0.1399, S_2_ = 0.1132, S_3_ = -0.1743, S_4_ = 0.0950, S_5_ = -0.0783	*A*_2_>*A*_4_>*A*_5_>*A*_1_>*A*_3_
*λ* = 4	S_1_ = -0.0802, S_2_ = 0.1499, S_3_ = -0.1327, S_4_ = 0.1358, S_5_ = -0.0471	*A*_2_>*A*_4_>*A*_5_>*A*_1_>*A*_3_
*λ* = 5	S_1_ = -0.0410, S_2_ = 0.1708, S_3_ = -0.1070, S_4_ = 0.1569, S_5_ = -0.0294	*A*_2_>*A*_4_>*A*_5_>*A*_1_>*A*_3_
*λ* = 6	S_1_ = -0.0130, S_2_ = 0.1851, S_3_ = -0.0893, S_4_ = 0.1684, S_5_ = -0.0167	*A*_2_>*A*_4_>*A*_1_>*A*_5_>*A*_3_
*λ* = 10	S_1_ = 0.0489, S_2_ = 0.2157, S_3_ = -0.0534, S_4_ = 0.1871, S_5_ = 0.0155	*A*_2_>*A*_4_>*A*_1_>*A*_5_>*A*_3_
*λ* = 20	S_1_ = 0.1033, S_2_ = 0.2423, S_3_ = -0.0265, S_4_ = 0.1993, S_5_ = 0.0435	*A*_2_>*A*_4_>*A*_1_>*A*_5_>*A*_3_
*λ* = 50	S_1_ = 0.1370, S_2_ = 0.2587, S_3_ = -0.0107, S_4_ = 0.2057, S_5_ = 0.0595	*A*_2_>*A*_4_>*A*_1_>*A*_5_>*A*_3_
*λ* = 100	S_1_ = 0.1486, S_2_ = 0.2644, S_3_ = -0.0051, S_4_ = 0.2079, S_5_ = 0.0649	*A*_2_>*A*_4_>*A*_1_>*A*_5_>*A*_3_

### 5.3 Comparisons

In this subsection, seven existing representative MAGDM methods and the proposed MAGDM method are qualitatively and quantitatively compared to verify the feasibility and effectiveness of the proposed method. In addition, to verify the ability of the proposed method to reduce the negative effect of extreme attribute values, a comparative analysis considering the interrelationships among attributes is carried out.

#### 5.3.1 Qualitative comparison

In general, a qualitative comparison among different MAGDM methods can be carried out by comparing their characteristics. For the existing seven methods and the proposed method, the comparison characteristics selected are: whether information is expressed by *q*ROFNs, the flexibility in the aggregation of *q*ROFNs or IFNs, whether interrelationships of multiple attributes are considered, whether the partitioned input arguments are considered, and the ability of the method to reduce the negative influence of unduly high or unduly low attribute values on the aggregation results. The results of the comparison are shown in Table 10.

**Table 10:** The results of qualitative comparison.

Methods	Information by *q*ROFNs	Flexibility	Whether considers interrelationships of multiple attributes	Ability to reduce the negative effect	Whether considers the partitioned input arguments
IFWAHA [8]	No	Satisfactory	Yes	No	No
IFFPA [23]	No	Limited	Yes	Yes	No
*q*ROFWA [25]	Yes	Limited	No	No	No
*q*ROFWBM [26]	Yes	Limited	Yes	No	No
*q*ROFWPBM [28]	Yes	Limited	Yes	No	Yes
*q*ROFWGHM[37]	Yes	Limited	Yes	No	No
*q*ROFWPHM[38]	Yes	Limited	Yes	No	Yes
*q*ROFDWPPHM	Yes	Satisfactory	Yes	Yes	Yes

The information is expressed by *q*-rung orthopair fuzzy numbers, and the aggregation is based on the operation of the DTT family of ATT. Therefore, the feasible space of the proposed method is larger than that of the other methods, and the modeling of fuzzy and uncertain information is more flexible and accurate. The Heronian mean operator has been used to consider interrelationships among multiple attributes. The partitioned Heronian mean can model interrelationships among attributes more accurately than the Heronian mean operator due to the incorporation of the partitioned average operator, which can handle situations where there is no correlation between attributes. In addition, because the proposed method incorporates the PA operator, it has the ability to reduce the negative effect of extreme attribute values. To summarize the qualitative comparison above, the proposed method has desirable flexibility in both aggregating the *q*-rung orthopair fuzzy information and dealing with the interrelationships of attributes and has the ability to reduce the negative effect of the deviation in some attribute values.

#### 5.3.2 Quantitative comparison

In the following, to verify the effectiveness of the proposed method and to explore its advantages, seven representative methods are applied to the example in subsection 5.1 and compared with the proposed method. They are the IFWAHA, IFFPA, *q*ROFWA, *q*ROFWBM, *q*ROFWPBM, *q*ROFWGHM and *q*ROFWPHM methods. The comparison results are shown in Table 11 (suppose *λ* = 1.5, *q* = 2, *p* = 3, *a =* 1 and *b =* 2).

**Table 11:** The results of quantitative comparison.

Operator	Scores of the five alternatives	Ranking
IFWAHA [8]	S_1_ = 0.1800, S_2_ = 0.4040, S_3_ = 0.0880, S_4_ = 0.3030, S_5_ = 0.2800	*A*_2_>*A*_4_>*A*_5_>*A*_1_>*A*_3_
IFFPA [23]	S_1_ = 0.5570, S_2_ = 0.6860, S_3_ = 0.5180, S_4_ = 0.6390, S_5_ = 0.6120	*A*_2_>*A*_4_>*A*_5_>*A*_1_>*A*_3_
*q*ROFWA[25]	S_1_ = 0.0881, S_2_ = 0.3222, S_3_ = 0.0390, S_4_ = 0.2420 S_5_ = 0.2028	*A*_2_>*A*_4_>*A*_5_>*A*_1_>*A*_3_
*q*ROFWBM[26]	S_1_ = -0.5979, S_2_ = -0.4809, S_3_ = -0.6220, S_4_ = -0.5051, S_5_ = -0.5480	*A*_2_>*A*_4_>*A*_5_>*A*_1_>*A*_3_
*q*ROFWPBM[28]	S_1_ = -0.4927, S_2_ = -0.3397, S_3_ = -0.4857, S_4_ = -0.3194, S_5_ = -0.4073	*A*_4_>*A*_2_>*A*_5_>*A*_3_>*A*_1_
*q*ROFWGHM [37]	S_1_ = 0.2212, S_2_ = 0.4559, S_3_ = 0.1290, S_4_ = 0.3248, S_5_ = -0.2866	*A*_2_>*A*_4_>*A*_5_>*A*_1_>*A*_3_
*q*ROFWPHM [38]	S_1_ = -0.7695, S_2_ = -0.6971, S_3_ = -0.7700, S_4_ = -0.6711, S_5_ = -0.7176	*A*_4_>*A*_2_>*A*_5_>*A*_1_>*A*_3_
*q*ROFDWPPHM	S_1_ = -0.3465, S_2_ = -0.0490, S_3_ = -0.3294, S_4_ = -0.0642, S_5_ = -0.2566	*A*_2_>*A*_4_>*A*_5_>*A*_3_>*A*_1_

(1) Comparison with Liu and Chen’s method [8] based on the intuitionistic fuzzy weighted Archimedean Heronian aggregation (IFWAHA) operator: The proposed method obtains the same first three alternatives as Liu and Chen’s method, even though the rankings are slightly different. This shows the effectiveness and validity of the proposed method. In the following, the characteristics of the proposed method and of Liu and Chen’s method are compared; the characteristics being compared are the expressiveness of fuzzy information, whether the interrelationships among different attributes are considered, and whether the attitudes of the decision makers are considered.

1) Expressiveness: The proposed method is based on *q*ROFNs, whereas Liu and Chen’s method is based on IFNs, which are a special case of *q*ROFNs (*q* = 1). The expressiveness of fuzzy information of Liu and Chen’s method is limited to IFNs, whereas the proposed method can express fuzzy information more widely via assigning different values to *q*. Thus, the proposed method is more flexible for MAGDM problems.
2) Interrelationships: The proposed method is based on the PHM operator, whereas Liu and Chen’s method is based on the HM operator. Both the HM and PHM operators have the ability to describe the interrelationships among different attributes, but the PHM operator inherits all features of the HM operator and partitions attributes into different parts. In addition, the proposed method also uses the PA operator, which can reduce the influence of unreasonable data. Thus, the proposed method can obtain more reliable aggregation results via considering the interrelationships of attributes and partitioned attributes.
3) Attitudes: The decision maker's attitude usually has an important influence on the results of decision making. In Liu and Chen’s method, attitudes are reflected by a parameter (*λ*) in the Hamacher operator. As the value of *λ* increases, the attitude will shift from pessimistic to optimistic. Although *λ* can represent the attitudes of a decision maker, how to set a desirable value to *λ* is not specified. In the proposed method, 0 < *λ* ≤ 1 for pessimistic decision makers, 1< *λ* ≤5 for neutral decision makers, and *λ>*5 for optimistic decision makers. Thus, the proposed method can use different values of *λ* to set different levels for the attitudes of decision makers.

(2) Comparison with Zhang et al.’s method [23] based on the intuitionistic fuzzy frank power aggregation (IFFPA) operator: The proposed method obtains the same first three alternatives as Zhang et al.’s method, even though the rankings of *A*_1_ and *A*_3_ are opposite. In the following, the characteristics of the proposed method and Zhang et al.’s method are compared; the characteristics being compared are the expressiveness of fuzzy information and whether the interrelationships among different attributes are considered.

1) Expressiveness: As with the method in comparison (1), the expressiveness of fuzzy information of Zhang et al.’s method is limited to IFNs, whereas the proposed method can express fuzzy information more widely via assigning different values to *q*. Thus, the proposed method is more flexible for MAGDM problems.
2) Interrelationships: The proposed method is based on the PHM operator, which has the ability to describe the interrelationships among different attributes, and the PA operator, which can reduce the influence of unreasonable data and consider the relationships among the input values of attributes, whereas Zhang et al.’s method is based on the PA operator. Thus, the proposed method can obtain more reliable aggregation results by considering the interrelationships of attributes and partitioned attributes.
3) Comparison with Liu and Wang’s method [25] based on the *q*-rung orthopair fuzzy weighted averaging (*q*ROFWA) operator: It can be seen from Table 11 that the ranking results of both methods are the same except for the two alternatives ranked fourth and fifth. Although the two operators have the same results for this example, the *q*ROFWA operator can only perform simple weighted averaging operations on *q*ROFNs, and it does not consider the interrelationship among different input attribute values. As in the above example, attribute *C*_1_ is related to attribute *C*_2_, and attribute *C*_3_ is related to attribute *C*_4_. The proposed *q*ROFDWPPHM operator in the paper can reflect the interrelationships among different attributes, especially in the case of the combination of the PA and PHM operators. Thus, the proposed method is more reliable than Liu and Wang’s method because it considers the interrelationships of the different input arguments.
4) Comparison with Liu and Liu’s method [26] based on the *q*-rung orthopair fuzzy weighted Bonferroni mean (*q*ROFWBM) operator: It can be seen from Table 11 that the ranking results of both methods are the same except for two alternatives ranked fourth (*A*_1_) and fifth (*A*_3_). The *q*ROFWBM operator is extended from the BM operator to aggregate *q*ROFNs, whereas the proposed operator is based on the HM operator. Yu et al. [39] demonstrated that the HM operator has more advantages than the BM operator. In addition, the *q*ROFWBM operator assumes that each attribute is related to all the other attributes, which obviously is not the case in most real situations. In contrast with the proposed *q*ROFWBM operator, the proposed *q*ROFDWPPHM operator eliminates the effect of the association of unrelated attributes on aggregation and ordering results. Thus, the proposed method has more advantages than Liu and Liu’s method because it considers the irrelevant relationships between input arguments in the real situations.
5) Comparison with Yang and Pang’s method [28] based on the *q*-rung orthopair fuzzy weighted partitioned Bonferroni mean (*q*ROFWPBM) operator: As shown in Table 11, the ranking results of the alternatives obtained by Yang and Pang’s method are different from those obtained by the proposed method. The *q*ROFWPBM operator is extended from the PBM operator to aggregate *q*ROFNs, whereas the proposed operator is based on the PHM operator. Thus, the common feature of these two operators is that they can reflect the relationships between properties; in particular, the relationships between unrelated properties can be considered by partition. However, the operational rules of the proposed method are based on the DTT rather than on the simple operational rules of *q*ROFNs, and the proposed method is further extended from the PHM operator to the power partitioned Heronian mean operator by incorporating the PA operator. This is why the ranking results obtained by the *q*ROFWPBM operator are significantly different from those obtained by the *q*ROFDWPPHM operator. Thus, the proposed method is more suitable than Yang and Pang’s method for dealing with the example mentioned in this paper.
6) Comparison with Wei et al.’s method [37] based on *q*-rung orthopair fuzzy weighted geometric Heronian mean (*q*ROFWGHM) operator: As can be seen from Table 11, the proposed method obtains the same first three alternatives as Wei et al.’s method, even though the rankings of *A*_1_ and *A*_3_ are reversed. However, the GHM operator assumes that each attribute is related to all the other attributes and that there are some decision cases that do not satisfy this precondition. As in the above investment selection example, the attributes *C*_1_ (the risk analysis) and *C*_2_ (the growth analysis) have no relationship with the attributes *C*_3_ (the social-political impact analysis) and *C*_4_ (the environmental impact analysis). Thus, the proposed method is more suitable than Wei et al.’s method for dealing with MAGDM problems.
7) Comparison with Liu et al.’s method [38] based on *q*-rung orthopair fuzzy weighted partitioned Heronian mean (*q*ROFWPHM) operator: Table 11 shows that the proposed method obtains different results from Liu et al.’s method for the first two alternatives, while the other alternatives are the same. There are two reasons for this: operational rules and operators. First, Liu et al.’s method is based on simple operational rules of *q*ROFNs, whereas the operational rules of the proposed method are based on the DTT; the parameter *λ* makes the information aggregation flexible. Second, the proposed *q*ROFDWPPHM operator is based on the PHM operator and combined with the PA operator, whereas Liu et al.’s method is only based on the PHM operator. Thus, the proposed method is more powerful and flexible than Liu et al.’s method because it considers the interrelationships of the aggregated arguments.

#### 5.3.3 Further comparative analysis

In the previous subsection, the proposed method was compared with some current methods, and the advantages of the proposed method were analyzed. However, the advantages of the proposed method are not obvious since the ranking results are almost always the same. To show the advantages more intuitively, a further comparison is carried out. By modifying specific inputs to compare with the actual ranking in the example, it can be shown that the ranking result can be changed by reducing other evaluation values of alternative *A*_2_. For example, the values of *μ1 21* and *μ1 22* are modified from 0.7 to 0.01, and the values of *v1 21* and *v1 22* are reduced from 0.3 to 0.99. That is, the evaluation values of *A*_2_ with respect to the attributes *C*_1_ and *C*_2_ are reduced from (0.7,0.3) to (0.01,0.99). To clearly display the advantages of the proposed method, Liu et al.’s method [8] and Liu’s method [22] (both of which were also compared in [8]) are considered in this comparison. Liu et al.’s method and the proposed method are based on the HM operators, and Liu’s method is based on the Hamacher operators. The former two methods can consider interrelationships among attributes, while the latter method cannot. The scores and the ranking results of these three methods are shown in Tables 12 and 13 (suppose *λ* = 5, *q* = 2, *p* = 3, *a =* 1 and *b =* 2).

**Table 12:** The scores of the three methods.

($\Theta_{21}^{1},\Theta_{22}^{1}$)	The proposed method	Liu et al.’s method [8]	Liu’s method [22]
(0.7,0.3)	S_1_ = -0.0410, S_2_ = 0.1708, S_3_ = -0.1070, S_4_ = 0.1569, S_5_ = -0.0294	S_1_ = -0.091, S_2_ = 0.327, S_3_ = 0.023, S_4_ = 0.234, S_5_ = 0.184	S_1_ = 0.065, S_2_ = 0.354, S_3_ = -0.020, S_4_ = 0.227, S_5_ = 0.153
(0.6,0.4)	S_1_ = -0.0410, S_2_ = 0.1669, S_3_ = -0.1070, S_4_ = 0.1569, S_5_ = -0.0294	S_1_ = -0.091, S_2_ = 0.306, S_3_ = 0.023, S_4_ = 0.234, S_5_ = 0.184	S_1_ = 0.065, S_2_ = 0.333, S_3_ = -0.020, S_4_ = 0.227, S_5_ = 0.153
(0.5,0.5)	S_1_ = -0.0410, S_2_ = 0.1622, S_3_ = -0.1070, S_4_ = 0.1569, S_5_ = -0.0294	S_1_ = -0.091, S_2_ = 0.288, S_3_ = 0.023, S_4_ = 0.234, S_5_ = 0.184	S_1_ = 0.065, S_2_ = 0.309 S_3_ = -0.020, S_4_ = 0.227, S_5_ = 0.153
(0.4,0.6)	S_1_ = -0.0410, S_2_ = 0.1610, S_3_ = -0.1070, S_4_ = 0.1569, S_5_ = -0.0294	S_1_ = -0.091, S_2_ = 0.272, S_3_ = 0.023, S_4_ = 0.234, S_5_ = 0.184	S_1_ = 0.065, S_2_ = 0.281, S_3_ = -0.020, S_4_ = 0.227, S_5_ = 0.153
(0.3,0.7)	S_1_ = -0.0410, S_2_ = 0.1609, S_3_ = -0.1070, S_4_ = 0.1569, S_5_ = -0.0294	S_1_ = -0.091, S_2_ = 0.258, S_3_ = 0.023, S_4_ = 0.234, S_5_ = 0.184	S_1_ = 0.065, S_2_ = 0.247, S_3_ = -0.020, S_4_ = 0.227, S_5_ = 0.153
(0.2,0.8)	S_1_ = -0.0410, S_2_ = 0.1609, S_3_ = -0.1070, S_4_ = 0.1569, S_5_ = -0.0294	S_1_ = -0.091, S_2_ = 0.246, S_3_ = 0.023, S_4_ = 0.234, S_5_ = 0.184	S_1_ = 0.065, S_2_ = 0202, S_3_ = -0.020, S_4_ = 0.227, S_5_ = 0.153
(0.1,0.9)	S_1_ = -0.0410, S_2_ = 0.1608, S_3_ = -0.1070, S_4_ = 0.1569, S_5_ = -0.0294	S_1_ = -0.091, S_2_ = 0.236, S_3_ = 0.023, S_4_ = 0.234, S_5_ = 0.184	S_1_ = 0.065, S_2_ = 0.130, S_3_ = -0.020, S_4_ = 0.227, S_5_ = 0.153
(0.05,0.95)	S_1_ = -0.0410, S_2_ = 0.1608, S_3_ = -0.1070, S_4_ = 0.1569, S_5_ = -0.0294	S_1_ = -0.091, S_2_ = 0.232, S_3_ = 0.023, S_4_ = 0.234, S_5_ = 0.184	S_1_ = 0.065, S_2_ = 0.063, S_3_ = -0.020, S_4_ = 0.227, S_5_ = 0.153
(0.01,0.99)	S_1_ = -0.0410, S_2_ = 0.1608, S_3_ = -0.1070, S_4_ = 0.1569, S_5_ = -0.0294	S_1_ = -0.091, S_2_ = 0.228, S_3_ = 0.023, S_4_ = 0.234, S_5_ = 0.184	S_1_ = 0.065, S_2_ = -0.078, S_3_ = -0.020, S_4_ = 0.227, S_5_ = 0.153

**Table 13:** The ranking results of the three methods.

($\Theta_{21}^{1},\Theta_{22}^{1}$)	The proposed method	Liu et al.’s method [8]	Liu’s method [22]
(0.7,0.3)	*A*_2_>*A*_4_>*A*_5_>*A*_1_>*A*_3_	*A*_2_>*A*_4_>*A*_5_>*A*_1_>*A*_3_	*A*_2_>*A*_4_>*A*_5_>*A*_1_>*A*_3_
(0.6,0.4)	*A*_2_>*A*_4_>*A*_5_>*A*_1_>*A*_3_	*A*_2_>*A*_4_>*A*_5_>*A*_1_>*A*_3_	*A*_2_>*A*_4_>*A*_5_>*A*_1_>*A*_3_
(0.5,0.5)	*A*_2_>*A*_4_>*A*_5_>*A*_1_>*A*_3_	*A*_2_>*A*_4_>*A*_5_>*A*_1_>*A*_3_	*A*_2_>*A*_4_>*A*_5_>*A*_1_>*A*_3_
(0.4,0.6)	*A*_2_>*A*_4_>*A*_5_>*A*_1_>*A*_3_	*A*_2_>*A*_4_>*A*_5_>*A*_1_>*A*_3_	*A*_2_>*A*_4_>*A*_5_>*A*_1_>*A*_3_
(0.3,0.7)	*A*_2_>*A*_4_>*A*_5_>*A*_1_>*A*_3_	*A*_2_>*A*_4_>*A*_5_>*A*_1_>*A*_3_	*A*_2_>*A*_4_>*A*_5_>*A*_1_>*A*_3_
(0.2,0.8)	*A*_2_>*A*_4_>*A*_5_>*A*_1_>*A*_3_	*A*_2_>*A*_4_>*A*_5_>*A*_1_>*A*_3_	*A*_4_>*A*_2_>*A*_5_>*A*_1_>*A*_3_
(0.1,0.9)	*A*_2_>*A*_4_>*A*_5_>*A*_1_>*A*_3_	*A*_2_>*A*_4_>*A*_5_>*A*_1_>*A*_3_	*A*_4_>*A*_5_>*A*_2_>*A*_1_>*A*_3_
(0.05,0.95)	*A*_2_>*A*_4_>*A*_5_>*A*_1_>*A*_3_	*A*_4_>*A*_2_>*A*_5_>*A*_1_>*A*_3_	*A*_4_>*A*_5_>*A*_2_>*A*_1_>*A*_3_
(0.01,0.99)	*A*_2_>*A*_4_>*A*_5_>*A*_1_>*A*_3_	*A*_4_>*A*_2_>*A*_5_>*A*_1_>*A*_3_	*A*_4_>*A*_5_>*A*_1_>*A*_3_>*A*_2_

It can be seen from Table 12 that as the degree of membership decreases and the degree of nonmembership increases, the score S_2_ of alternative A_2_ decreases gradually, but the rate of decrease is different. Among all three methods, the reduction rate of the proposed method is the slowest, and the total reduction is only 0.01, while Liu et al.'s method has an amplitude of 0.099 and an amplitude of 0.432. This can be explained by the interrelationships among attributes. The proposed method is based on the PA operator and the PHM operator, which can take into account the relationships of the input data and classify the data in accordance with the correlations between the attributes, while Liu's method only uses the HM operator. Although the correlation between attributes can also be considered, it is not sufficiently comprehensive, and Liu's method does not consider the correlation. This shows that the proposed method is more reasonable than the other two methods, especially in an actual situation. For various reasons, decision makers may provide some unduly high or unduly low evaluation values, and the proposed operator can well reduce such a negative impact.

As can be seen from Table 13, the ranking results of the proposed method remain the same as the attribute values change. The ranking given by Liu et al.'s method starts to change when the attribute value changes to (0.05,0.95), and the ranking of alternatives *A*_2_ and *A*_4_ are reversed. Liu's method starts to change when the attribute value changes to (0.2,0.8). It can be inferred intuitively from Tables 12 and 13 that when the attribute values in the fuzzy matrix of decision maker *D*_1_ are changed from (0.7,0.3) to (0.01,0.99) and the corresponding attribute values of the other two matrices are (0.5,0.4) and (0.5,0.3), this set of fuzzy numbers is unreasonable. It is further demonstrated that the other two methods are greatly affected by unreasonable data, while the proposed method has a strong ability to process unreasonable input data; that is, the proposed *q*ROFDPPHM operator can consider the interrelationships among attribute values and reduce the negative influence of biased attribute values.

## 6. Conclusions

In this paper, a set of *q*-rung orthopair fuzzy operational rules is developed based on the Dombi t-conorm and t-norm. Then, a *q*ROFDPHM operator and a *q*ROFDWPHM operator are presented. To reduce the negative impact of unreasonable attribute values on aggregated results, a *q*ROFDPPHM operator and a *q*ROFDWPPHM operator are presented via combining the PHM operator with the PA operator based on *q*ROFSs. Moreover, a MAGDM method based on the proposed operators is also proposed. A practical example and a set of experiments are provided to illustrate the proposed approach. A set of comparisons is performed to demonstrate the effectiveness and feasibility of the proposed approach. The results of the experiments and comparisons show that the proposed method is feasible, effective, and flexible. Compared with the existing methods, the proposed method has the following advantages:

(1) It can consider the interrelationships of aggregated arguments;
(2) It has desirable flexibility in aggregating the q-rung orthopair fuzzy information;
(3) It can reduce the negative impact of unreasonable attribute values on aggregated results.

In future studies, other new types of ATT will be studied and extended to the power partitioned Heronian aggregation operator based on *q*ROFNs. In addition, the proposed operators and method will be applied to some practical decision-making problems, such as recommendation systems, performance evaluations, supplier selection evaluations and pattern recognition systems.

## Appendix A. Proofs of Eqs (15)–(20)

### Proof

Let Θ = (*μ*, *v*), Θ_1_ = (*μ*_1_, *v*_1_) and Θ_2_ = (*μ*_2_, *v*_2_). From (11), we have:

$$\Theta_{1}⊕\Theta_{2}=\left(g^{-1}(g(\mu_{1})+g(\mu_{2}),f^{-1}(f(v_{1})+f(v_{2})\right)$$

$$\Theta_{2}⊕\Theta_{1}=\left(g^{-1}(g(\mu_{2})+g(\mu_{1}),f^{-1}(f(v_{2})+f(v_{1})\right).$$

Then, Θ_1_⊕Θ_2_ = Θ_2_⊕Θ_1_.

Thus, the proof of Eq (15) is completed.

According to (12), we have:

$$\Theta_{1}⊗\Theta_{2}=\left(f^{-1}(f(\mu_{1})+f(\mu_{2})),g^{-1}(g(v_{1})+g(v_{2}))\right)$$

$$\Theta_{2}⊗\Theta_{1}=\left(f^{-1}(f(\mu_{2})+f(\mu_{1})),g^{-1}(g(v_{2})+g(v_{1}))\right)$$

Then, Θ_1_⊗Θ_2_ = Θ_2_⊗Θ_1_.

Thus, the proof of Eq (16) is completed.

According to (11) and (13), we have:

$$\begin{array}{l} \delta (\Theta_{1}⊕\Theta_{2})=\delta \left(g^{-1}(g(\mu_{1})+g(\mu_{2})),f^{-1}(f(v_{1})+f(v_{2}))\right)\\ \;=\left(g^{-1}(\delta (g(\mu_{1})+g(\mu_{2}))),f^{-1}(\delta (f(v_{1})+f(v_{2})))\right) \end{array},$$

$$\delta \Theta_{1}=\left(g^{-1}(\delta g(\mu_{1})),f^{-1}(\delta f(v_{1}))\right),$$

$$\delta \Theta_{2}=\left(g^{-1}(\delta g(\mu_{2})),f^{-1}(\delta f(v_{2}))\right),$$

$$\delta \Theta_{1}⊕\delta \Theta_{2}=\left(g^{-1}(\delta (g(\mu_{1})+g(\mu_{2}))),f^{-1}(\delta (f(v_{1})+f(v_{2})))\right),$$

$$\delta \Theta =\left(g^{-1}(\delta g(\mu )),f^{-1}(\delta f(v))\right),$$

$$\tau \Theta =\left(g^{-1}(\tau g(\mu )),f^{-1}(\tau f(v))\right),$$

$$\delta \Theta ⊕\tau \Theta =\left(g^{-1}(\delta g(\mu )+\tau g(\mu )),f^{-1}(\delta f(v)+\tau f(v))\right),$$

and

$$\begin{array}{l} (\delta +\tau )\Theta =\left(g^{-1}((\delta +\tau )g(\mu )),f^{-1}((\delta +\tau )f(v))\right)\\ \;=\left(g^{-1}(\delta g(\mu )+\tau g(\mu )),f^{-1}(\delta f(v)+\tau f(v))\right) \end{array}.$$

Thus, *δ*(Θ_1_⊕Θ_2_) = *δ*(Θ_1_⊕ *δ*(Θ_2_ and *δ*Θ⊕τΘ = (*δ*+*τ*)Θ.

Thus, the proofs of Eqs (17) and (18) are completed.

According to (12) and (14), we have:

$$\Theta_{1}{}^{\delta }=\left(f^{-1}(\delta f(\mu_{1})),g^{-1}(\delta g(v_{1}))\right),$$

$$\Theta_{2}{}^{\delta }=\left(f^{-1}(\delta f(\mu_{2})),g^{-1}(\delta g(v_{2}))\right),$$

$$\Theta_{1}{}^{\delta }⊗\Theta_{2}{}^{\delta }=\left(f^{-1}(\delta f(\mu_{1})+\delta f(\mu_{2})),g^{-1}(\delta g(v_{1})+\delta g(v_{2}))\right),$$

and

$$\begin{array}{l} {\left(\Theta_{1}⊗\Theta_{2}\right)}^{\delta }=\left(f^{-1}(\delta (f(\mu_{1})+f(\mu_{2}))),g^{-1}(\delta (g(v_{1})+g(v_{2})))\right)\\ \;=\left(f^{-1}(\delta f(\mu_{1})+\delta f(\mu_{2})),g^{-1}(\delta g(v_{1})+\delta g(v_{2}))\right) \end{array}.$$

Then, $\Theta_{{}_{1}}^{\delta }⊗\Theta_{{}_{2}}^{\delta }={\left(\Theta_{1}⊗\Theta_{2}\right)}^{\delta }$.

Therefore,

$$\Theta^{\delta }=\left(f^{-1}(\delta f(\mu )),g^{-1}(\delta g(v))\right),$$

$$\Theta^{\tau }=\left(f^{-1}(\tau f(\mu )),g^{-1}(\tau g(v))\right)$$

Thereafter,

$$\Theta^{\tau +\delta }=\left(f^{-1}((\tau +\delta )f(\mu )),g^{-1}((\tau +\delta )g(v))\right),$$

and

$$\begin{array}{l} \Theta^{\delta }⊗\Theta^{\tau }=\left(f^{-1}(\delta f(\mu )+\tau f(\mu )),g^{-1}(\delta g(v)+\tau g(\mu ))\right)\\ \;=\left(f^{-1}((\delta +\tau )f(\mu )),g^{-1}((\delta +\tau )g(v))\right) \end{array}$$

Then, Θ^*δ*^⊗Θ^*τ*^ = Θ^*τ*+*δ*^.

Thus, the proofs of Eqs (19) and (20) are completed.

## Appendix B. Proof of Theorem 1

**Proof**.

According to Definition 6, we have:

Θhia=((11+(a(1−μhiqμhiq)λ)1λ)1q,(1−11+(a(vhiq1−vhiq)λ)1λ)1q)=((11+a1λ(1−μhiqμhiq))1q,(1−11+a1λ(vhiq1−vhiq))1q)

Θhjb=((11+(b(1−μhjqμhjq)λ)1λ)1q,(1−11+(b(vhjq1−vhjq)λ)1λ)1q)=((11+b1λ(1−μhjqμhjq)λ)1q,(1−11+b1λ(vhjq1−vhjq))1q)

Let 1−μhiqμhiq=f(μhi)1λ, 1−μhjqμhjq=f(μhj)1λ, vhiq1−vhiq=g(vhi)1λ, and vhjq1−vhjq=g(vhj)1λ; then

Θhia=((11+(af(μhi))1λ)1q,(1−11+(ag(vhi))1λ)1q)

Θhjb=((11+(bf(μhj))1λ)1q,(1−11+(bg(vhj))1λ)1q)

Θhia⊗Θhjb=((11+(af(μhi)+bf(μhj))1λ)1q,(1−11+(ag(vhi)+bg(vhj))1λ)1q)

⊕j=i|Ph|Θhia⊗Θhjb=((1−11+(∑j=i|Ph|1(af(μhi)+zf(μhj)))1λ)1q,(11+(∑j=i|Ph|1(ag(vhi)+bg(vhj)))1λ)1q)

⊕i=1|Ph|⊕j=i|Ph|Θhia⊗Θhjb=((1−11+(∑i=1|Ph|∑j=i|Ph|1(af(μhi)+bf(μhj)))1λ)1q,(11+(∑i=1|Ph|∑j=i|Ph|1(ag(vhi)+bg(vhj)))1λ)1q)

2|ph|(|ph|+1)⊕i=1|Ph|⊕j=i|Ph|Θhia⊗Θhjb=((1−11+(2|ph|(|ph|+1)∑i=1|Ph|∑j=i|Ph|1(af(μhi)+bf(μhj)))1λ)1q,(11+(2|ph|(|ph|+1)∑i=1|Ph|∑j=i|Ph|1(ag(vhi)+bg(vhj)))1λ)1q)

⊕h=1d(2|ph|(|ph|+1)⊕i=1|Ph|⊕j=i|Ph|Θhia⊗Θhjb)=((1−11+(∑h=1d(2|ph|(|ph|+1)∑i=1|Ph|∑j=i|Ph|1(af(μhi)+bf(μhj))))1λ)1q,(11+(∑h=1d(2|ph|(|ph|+1)∑i=1|Ph|∑j=i|Ph|1(ag(vhi)+bg(vhj))))1λ)1q)

(⊕h=1d(2|ph|(|ph|+1)⊕i=1|Ph|⊕j=i|Ph|Θhia⊗Θhjb))1a+b=((1/(1+(1/∑h=1d(2(a+b)|ph|(|ph|+1)∑i=1|Ph|∑j=i|Ph|1(af(μhi)+bf(μhj))))1λ))1q,(1−(1/(1+(1/∑h=1d(2(a+b)|ph|(|ph|+1)∑i=1|Ph|∑j=i|Ph|1(ag(vhi)+bg(vhj))))1λ)))1q)

1d(⊕h=1d(2|ph|(|ph|+1)⊕i=1|Ph|⊕j=i|Ph|Θhia⊗Θhjb))1a+b=((1−(1/(1+(1d∑h=1d(2(a+b)|ph|(|ph|+1)∑i=1|Ph|∑j=i|Ph|1(af(uhi)+bf(uhj))))1λ)))1q,(1/(1+(1d∑h=1d(2(a+b)|ph|(|ph|+1)∑i=1|Ph|∑j=i|Ph|1(ag(vhi)+bg(vhj))))1λ))1q)

It follows that $qROFDPHM^{a,b}(\Theta_{1},\Theta_{2},\ldots ,\Theta_{n})=\frac{1}{d}{\left(\overset{d}{\underset{h=1}{⊕}}\left(\frac{2}{\left|P_{h}\right|(\left|P_{h}\right|+1)}\overset{\left|P_{h}\right|}{\underset{i=1}{⊕}}\overset{\left|P_{h}\right|}{\underset{j=i}{⊕}}\Theta_{hi}^{a}⊗\Theta_{hj}^{b}\right)\right)}^{\frac{1}{a+b}}$ Thus, the proof of Theorem 1 is completed.

## Appendix C. Proof of Theorem 5

**Proof**.

According to Definition 6, we have:

whiΘhi=(1−11+(whi(uhiq1−uhiq)λ)1λ,11+(whi(1−vhiqvhiq)λ)1λ)1q=(11+whi1λ(μhiq1−μhiq),1−11+whi1λ(1−vhiqvhiq))1q

whjΘhj=(1−11+(whj(uhjq1−uhjq)λ)1λ,11+(whj(1−vhjqvhjq)λ)1λ,)1q=(11+whj1λ(μhjq1−μhjq),1−11+whi1λ(1−vhjqvhjq))1q

Let 1−μhiqμhiq=f(μhi)1λ, 1−μhjqμhjq=f(μhj)1λ, vhiq1−vhiq=g(vhi)1λ, and vhjq1−vhjq=g(vhj)1λ; then

(whiΘhi)a=((11+(a/whif(μhi))1λ)1q,(1−11+(a/whig(vhi))1λ)1q)

(whjΘhj)b=((11+(b/whjf(μhj))1λ)1q,(1−11+(b/whjg(vhj))1λ)1q)Therefore,

(whiΘhi)a⊗(whjΘhj)b=((11+(a/whif(μhi)+b/whjf(μhj))1λ)1q,(1−11+(a/whig(vhi)+b/whjg(vhj))1λ)1q)

⊕j=i|Ph|(whiΘhi)a⊗(whjΘhj)b=((1−11+(∑j=i|Ph|1(a/whif(μhi)+b/whjf(μhj)))1λ)1q,(11+(∑j=i|Ph|1(a/whig(vhi)+b/whjg(vhj)))1λ)1q)

⊕i=1|Ph|⊕j=i|Ph|(whiΘhi)a⊗(whjΘhj)b=((1−11+(∑i=1|Ph|∑j=i|Ph|1(a/whif(μhi)+b/whjf(μhj)))1λ)1q,(11+(∑i=1|Ph|∑j=i|Ph|1(a/whig(vhi)+b/whjg(vhj)))1λ)1q)

2|ph|(|ph|+1)⊕i=1|Ph|⊕j=i|Ph|(whiΘhi)a⊗(whjΘhj)b=((1−11+(2|ph|(|ph|+1)∑i=1|Ph|∑j=i|Ph|1(a/whif(μhi)+b/whjf(μhj)))1λ)1q,(11+(2|ph|(|ph|+1)∑i=1|Ph|∑j=i|Ph|1(a/whig(vhi)+b/whjg(vhj)))1λ)1q)

⊕h=1d(2|ph|(|ph|+1)⊕i=1|Ph|⊕j=i|Ph|(whiΘhi)a⊗(whjΘhj)b)=((1−11+(∑h=1d(2|ph|(|ph|+1)∑i=1|Ph|∑j=i|Ph|1(a/whif(μhi)+b/whjf(μhj))))1λ)1q,(11+(∑h=1d(2|ph|(|ph|+1)∑i=1|Ph|∑j=i|Ph|1(a/whig(vhi)+b/whjg(vhj))))1λ)1q)

(⊕h=1d(2|ph|(|ph|+1)⊕i=1|Ph|⊕j=i|Ph|(whiΘhi)a⊗(whjΘhj)b))1a+b=((1/(1+(1/∑h=1d(2(a+b)|ph|(|ph|+1)∑i=1|Ph|∑j=i|Ph|1(a/whif(μhi)+b/whjf(μhj))))1λ))1q,(1−(1/(1+(1/∑h=1d(2(a+b)|ph|(|ph|+1)∑i=1|Ph|∑j=i|Ph|1(a/whig(vhi)+b/whjg(vhj))))1λ)))1q)

1d(⊕h=1d(2|ph|(|ph|+1)⊕i=1|Ph|⊕j=i|Ph|(whiΘhi)a⊗(whjΘhj)b))1a+b=((1−(1/(1+(1d∑h=1d(2(a+b)|ph|(|ph|+1)∑i=1|Ph|∑j=i|Ph|1(a/(whif(uhi))+b/(whjf(uhj)))))1λ)))1q,(1/(1+(1d∑h=1d(2(a+b)|ph|(|ph|+1)∑i=1|Ph|∑j=i|Ph|1(a/(whig(vhi))+b/(whig(vhj)))))1λ))1q)

It follows that

$$qROFDWPHM^{a,b}(\Theta_{1},\Theta_{2},\ldots ,\Theta_{n})=\frac{1}{d}{\left(\overset{d}{\underset{h=1}{⊕}}\left(\frac{2}{\left|P_{h}\right|(\left|P_{h}\right|+1)}\overset{\left|P_{h}\right|}{\underset{i=1}{⊕}}\overset{\left|P_{h}\right|}{\underset{j=i}{⊕}}{\left(w_{hi}\Theta_{hi}^{}\right)}^{a}⊗{\left(w_{hj}\Theta_{hj}^{}\right)}^{b}\right)\right)}^{\frac{1}{a+b}}$$

Thus, the proof of Theorem 5 is completed.

## Supporting information

S1 File The code implementation of the method and the comparison method. (RAR)
